# Primary mapping of quantitative trait loci regulating multivariate horticultural phenotypes of watermelon (*Citrullus lanatus* L.)

**DOI:** 10.3389/fpls.2022.1034952

**Published:** 2023-01-12

**Authors:** Sikandar Amanullah, Shenglong Li, Benjamin Agyei Osae, Tiantian Yang, Farhat Abbas, Meiling Gao, Xuezheng Wang, Hongyu Liu, Peng Gao, Feishi Luan

**Affiliations:** ^1^ College of Horticulture and Landscape Architecture, Northeast Agricultural University, Harbin, China; ^2^ Key Laboratory of Biology and Genetic Improvement of Horticulture Crops (Northeast Region), Ministry of Agriculture and Rural Affairs, Harbin, China; ^3^ College of Horticulture, South China Agricultural University, Guangzhou, China; ^4^ College of Life Sciences, Agriculture and Forestry, Qiqihar University, Qiqihar, China

**Keywords:** watermelon (*Citrullus lanatus* L.), ovary, fruit, seed, genetic markers, QTL

## Abstract

Watermelon fruits exhibit a remarkable diversity of important horticultural phenotypes. In this study, we initiated a primary quantitative trait loci (QTL) mapping to identify the candidate regions controlling the ovary, fruit, and seed phenotypes. Whole genome sequencing (WGS) was carried out for two differentiated watermelon lines, and 350 Mb (96%) and 354 Mb (97%) of re-sequenced reads covered the reference *de novo* genome assembly, individually. A total of 45.53% non-synonymous single nucleotide polymorphism (nsSNPs) and 54.47% synonymous SNPs (sSNPs) were spotted, which produced 210 sets of novel SNP-based cleaved amplified polymorphism sequence (CAPS) markers by depicting 46.25% co-dominant polymorphism among parent lines and offspring. A biparental F_2:3_ mapping population comprised of 100 families was used for trait phenotyping and CAPS genotyping, respectively. The constructed genetic map spanned a total of 2,398.40 centimorgans (cM) in length and averaged 11.42 cM, with 95.99% genome collinearity. A total of 33 QTLs were identified at different genetic positions across the eight chromosomes of watermelon (Chr-01, Chr-02, Chr-04, Chr-05, Chr-06, Chr-07, Chr-10, and Chr-11); among them, eight QTLs of the ovary, sixteen QTLs of the fruit, and nine QTLs of the seed related phenotypes were classified with 5.32–25.99% phenotypic variance explained (PVE). However, twenty-four QTLs were identified as major-effect and nine QTLs were mapped as minor-effect QTLs across the flanking regions of CAPS markers. Some QTLs were exhibited as tightly localized across the nearby genetic regions and explained the pleiotropic effects of multigenic nature. The flanking QTL markers also depicted significant allele specific contributions and accountable genes were predicted for respective traits. Gene Ontology (GO) functional enrichment was categorized in molecular function (MF), cellular components (CC), and biological process (BP); however, Kyoto Encyclopedia of Genes and Genomes (KEGG) pathways were classified into three main classes of metabolism, genetic information processing, and brite hierarchies. The principal component analysis (PCA) of multivariate phenotypes widely demonstrated the major variability, consistent with the identified QTL regions. In short, we assumed that our identified QTL regions provide valuable genetic insights regarding the watermelon phenotypes and fine genetic mapping could be used to confirm them.

## Introduction

Watermelon (*Citrullus lanatus* L.) is an annual fruit crop that belongs to the Cucurbitaceae family, and it is a highly cultivated fruit with more than a thousand varieties (Pan et al., 2020).

Plants are mostly grown in tropical to temperate climates under high temperature requirements of about >25°C (77°F) to thrive (Walters et al., 2021). China is the major consumer and producer of watermelon, and the cultivation area accounts for nearly half (46.04%) of the world’s watermelon planting area. Among the world’s watermelon producing countries, China ranks first with 60 million tons production (Grumet et al., 2021).

The better quality and sweeter tasting watermelons are not only the result of natural selection (Chomicki and Renner, 2015), but they also depend upon artificial selection during their adaptation to the diverse environments (Pan et al., 2020). Since the start of watermelon domestication, the cultivated watermelons (citron, dessert, and egusi) have been classified as sub-species of the main species (*Citrullus lanatus*) (Fursa, 1972), exhibiting remarkable phenotypic diversity (Sandlin et al., 2012). Although there is some cross-ability among them, the genome dataset recommends their separation into three dissimilar species (Renner et al., 2014): (1) *C. lanatus* (Thunb.) Matsum. & Nakai is the dessert watermelon (also known as *C. lanatus* subsp. *vulgaris*), (2) C. *amarus* Schrad. is the citron watermelon (also known as *C. lanatus* subsp. *lanatus*), and (3) *C. mucosospermus* Fursa is the egusi watermelon (also called as *C. lanatus* subsp. *mucosospermus*) (Paris, 2015).

Watermelon genotypes have short and long ovaries with differentiated weight (Weetman, 1937; Osae et al., 2022), and flowers of some botanical varieties are monoecious or andromonoecious (Poole and Grimball, 1938; Aguado et al., 2020). The developed ovary and mature fruit display a high correlation since pre-anthesis. However, the obvious structure of mature fruit is reflected by gradual cell division and cell size elongation during each developmental stage (McKay, 1936; Weetman, 1937; Dou et al., 2018b). Fruit size and shape indexes vary within elongated, blocky, and rounded fruits effectively classified by representing a highly quantitative genetic architecture regulated by the contribution of polygenic architecture of allelic variants (Wehner et al., 2001; Gusmini and Wehner, 2006; Gusmini and Wehner, 2007; Sandlin et al., 2012; Kim et al., 2015).

Further, watermelon fruit-related quality traits are extremely connected with each other and significantly fascinate the consumer’s attention. Fruit weight varies from 1 kilogram (kg) to more than 10 kg and mainly depends upon the fruit size and shape, affecting the total crop yield (Gusmini and Wehner, 2006; Osae et al., 2022). Fruit flesh firmness is a standard quantitative trait that determines the edible quality of watermelon, and variations are genetically inherited with genotypes and environments. It is jointly regulated through polygenes and multifarious metabolic networks (Zhu et al., 2017) and the flesh cell size is increased by enhanced vacuolation at the initial days after pollination (DAP) (Sun et al., 2020). Fruit rind texture is highly diversified in numerous cultivars, which explains the good relationship with postharvest life. Watermelon cultivars with high rind-hardness are less prone to cracking and have better resistance to long-term storage (Liao et al., 2019; Yang et al., 2021). It was also shown that flesh firmness variation is an uneven and multifaceted phenomenon that is particularly shifted through inherited genetics (Khadivi-Khub, 2015), and endogenous lignin accumulation in peel stone cells form the hard ultrastructure of rind (Gao et al., 2013; Yang et al., 2022). An ethylene bio-synthesis related transcription factors “Md-ACO1, Md-ACS1, and MADS-box” contributed in the fruit ripening stages, nutrient metabolism, and hormone signal transduction, that mainly trigger the internal respiratory mechanism and led to a low firmness level in the fruit (Costa et al., 2005; Costa et al., 2008; Moreno et al., 2008; Costa et al., 2010; Vegas et al., 2013; Liao et al., 2019).

Fruit rind appearance can be gray, striped, and solid, or can be light-green, medium green, or dark green (Liang et al., 2022). Rind stries pattern can be blotchy, wavy, narrow, medium, or wide, depending on their presence on the rind surface (Zhang et al., 2018; Maragal et al., 2022). Multiple genetic basis of watermelon rind stripe pattern and rind color have been observed through differential gene architecture (Guner and Wehner, 2004; Dou et al., 2018a; Liu et al., 2020; Wang et al., 2022). Furthermore, the watermelon flesh color is a primary determinant of edible quality and is concerned with the carotenoids accumulation in internal chromoplast cells (Tadmor et al., 2005; Fang et al., 2020). Many accessions have different flesh color gradients, e.g., white, salmon-yellow, orange, red, canary yellow, pale green, and are thought to be polygenic (Gusmini and Wehner, 2006; Liu et al., 2015; Pei et al., 2021; Liang et al., 2022). The salmon-yellow color of the flesh is mainly developed by accumulation of pro-lycopene (tetra-cis-lycopene); orange develops from pro-lycopene and rarely from *β*-carotene, red from lycopene; and canary yellow watermelon results from accumulation of small amounts of xanthophylls and *β*-carotene (Tadmor et al., 2005; Bang et al., 2010; Branham et al., 2017; Subburaj et al., 2019). The natural variation in carotenoid accumulation takes place among the heirloom and exotic watermelon accessions, which induce an extensive range of flesh colors regulated by multiple genes (Fang et al., 2022).

Seed is an integral part of the plant life cycle that determines the vigorous growth and development of crop plants (Xiang et al., 2002). The size and shape of seed greatly vary in different crops; however, these were always considered as primary target for breeding selection throughout domestication (Gómez, 2004; Moles et al., 2005; Xia et al., 2018). The wild-type watermelons usually bear small and rounded-shaped seeds, while the improved cultivars bear much larger and variegated-shaped seeds (Guo et al., 2020). Most of the variations in seed size and shape, seed oil content, seed coat thickness, seed weight, and seed thickness are the effective outcomes of natural and artificial selection during adaptation to different environmental localities (Meru and McGregor, 2013; Guo et al., 2020), and are thought to be influenced by polygenic alleles in a moderate-type dominance fashion (Baboli and Kordi, 2010; Tian et al., 2012). However, the genetic basis of seed-related content is less known in fruit and vegetable crops and still needs much more attention for an in-depth understanding.

The primary mapping of multifaceted QTLs is a traditional molecular technique that has been well-employed for the identification of candidate genomic regions controlling various crop traits based on different types of genetic markers (Olsen and Wendel, 2013). Different generations of DNA-based genetic markers (RFLPs, RAPDs, AFLPs, SSRs, SNPs, and CAPSs) and derived mapping populations have been introduced and utilized for effective genetic mapping of major loci controlling watermelon traits, respectively (Hashizume et al., 1996; Levi et al., 2001; Levi et al., 2006). Whole genome sequencing approach coupled with the published *de novo* reference genome assembly of watermelon has assisted in developing the base-by-base SNP markers and high-resolution genetic linkage mapping (Guo et al., 2013). In recent years, SNP based codominant CAPS markers have been emerged as an efficient DNA markers for dissecting the major-effect QTLs of regulating the important qualitative and quantitative traits of melon and watermelon (Bang et al., 2007; Liu et al., 2015; Cheng et al., 2016; Liu et al., 2016; Li et al., 2017; Zhang et al., 2018; Liu et al., 2019; Luan et al., 2019; Wang et al., 2019; Sun et al., 2020; Amanullah et al., 2020; Amanullah et al., 2021; Zhang et al., 2021; Pei et al., 2021; Osae et al., 2021; Yang et al., 2021; Amanullah et al., 2022; Osae et al., 2022; Lv et al., 2022).

Until now, many researchers have performed significant molecular mapping studies, but the detailed genetic basis of many unexplored cultivars has not been completely resolved. The current study was aimed at primary mapping of novel genetic regions controlling watermelon ovary, fruit, and seed phenotypes by using novel SNP-derived CAPS markers and genetic segregation analysis in a developed biparental F_2:3_ mapping population.

## Materials and methods

### Plant materials and mapping population

**Figure 1:**
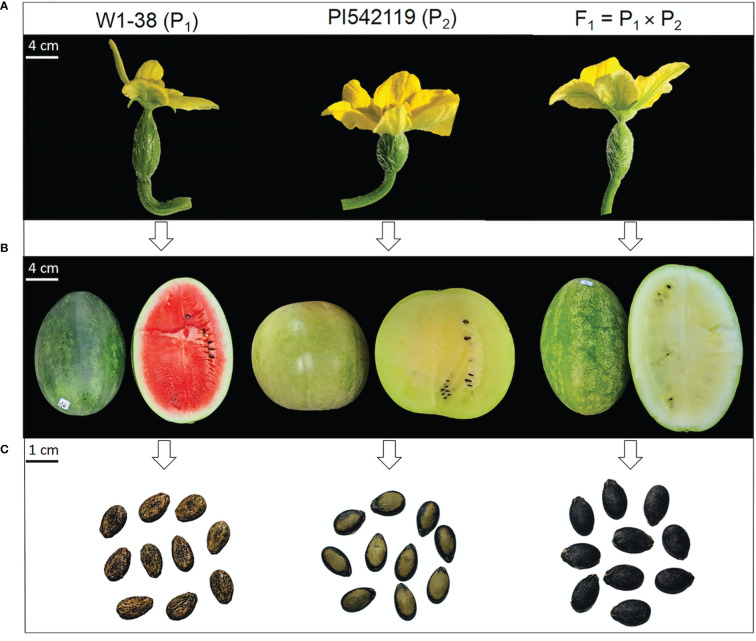
Primary phenotypes of comparative watermelon parent lines and developed F1 off-spring. **(A)** Ovary traits. **(B)** Fruit traits. **(C)** Seed traits, respectively.

Two comparative watermelon parent lines “W1-38 as P_1_ (cultivated female parent line*, Citrullus lanatus* L.) and PI542119 as P_2_ (male parent line, forage watermelon, *Citrullus* var. citroides)” were particularly chosen as experimental materials based on the primarily distinguished ovary, fruit, and seed phenotypes (**Figure 1**, **Supplementary Table S1**). These two parent lines were then crossed to develop their F_1_ offspring and F_2:3_ mapping population, respectively. A field experiment was carried out in a big plastic greenhouse at XiangYang Agricultural Farm of Northeast Agricultural University, Harbin, China.

In the first year of the experiment, plants of both parent materials were cultivated, total genomic DNA (GDNA) was extracted, whole genome sequencing was performed, and newly exported SNP-CAPS markers were validated for molecular genotyping, respectively. The raw data of DNA sequencing (DNA-seq) was uploaded to the online Sequence Read Archive (SRA) database (Accession: PRJNA878948, containing 2 biosamples) of the National Center for Biotechnology Information (NCBI). In the second year of experiment, seedlings of P_1_ (15-plants), P_2_ (15-plants), F_1_ (15-plants), and 100 F_2:3_ mapping families (5-plants of each family) were raised and one month old seedlings (true-leaves stages) were shifted into greenhouse. To ensure the healthy growth of plants, a mixture of 60% loamy soil, 30% compost, and 10% potting mixture (peat moss, perlite, and vermiculite) was used in the raised beds of the greenhouse. All the plants of the respective mapping families were grown by maintaining the row spacing (60 cm) and plant spacing (70 cm) in the planting geometry of a complete randomized block design (CRBD), and common horticultural techniques were subsequently applied, respectively.

### Evaluation of phenotypes

The ovary, fruit, and seed phenotypes of P_1_, P_2_, F_1_, and F_2:3_ mapping population were evaluated as earlier reported method (Pei et al., 2021; Liang et al., 2022; Maragal et al., 2022; Osae et al., 2022). For the ovary phenotypes, the anthesis period of each plant was observed on a daily basis, then fresh ovaries were plucked just before the day of flower opening. Ovary weight (OWt) was freshly weighed in grams (gm), ovary length (OL) was measured from the proximal end to the distal end, and ovary width (OW) was checked at the widest part from one edge to another edge. OL and OW were measured in millimeter (mm) and ovary shape index (OSI) was the ratio of ovary length to width (OL/OW).

For the fruit phenotypes, flowers were pollinated and mature watermelon fruits were harvested between 35 and 45 days after pollination (DAP). One fruit from each plant was harvested and fruit weight (FWt) was freshly weighed in kilogram (kg) units by using a portable digital weighing machine having high measuring accuracy. Fruit length (FL) was measured from top (stem end to lower end) to bottom (lower end) section, fruit width (FW) was checked from equatorial fruit diameter in centimeter (cm) units, and fruit shape index (FSI) was evaluated by the ratio of FL/FW. Fruit rind thickness (FRT) was checked by observing the distance between exocarp and mesocarp layers and recorded in millimeter (mm) units. Fruit flesh firmness (FFF) was tested by operating the digital fruit firmness tester (FR-5120) having a high-precision sensor with peak hold. The tip of the meter was steeply inserted at five different points of flesh (right, left, mid-point, top, bottom) and the firmness indicator mean was expressed in kg/cm^2^. Brix of fruits (FBR) was quantified from the squeezed juice samples and checked in percentage (%) using the hand-held refractometer (Atago Co., Ltd., Tokyo, Japan). The qualitative fruit traits were observed based on their visual phenotypes. Fruit rind color (FRC) was checked by observing the outer rind surface color “dark-green (DG), light-green (LG), and intermediate (I)” and scoring as 3 (DG), 1 (LG), and 2 (I). Fruit rind stripes (FRS) were observed based on their pattern differences on rind surfaces as “striped wavy (W) and non-striped blotchy (B)”, and visually scored as 0 (W), 1 (B). Fruit flesh color (FFC) was judged by the naked eye using the differentiated color scoring rate of “(3) red (R), (1) pale green (PG), and (2) yellow (Y)”, respectively.

For the seed phenotypes, seeds were extracted from the flesh cavity of each harvested fruit, then gently washed, and dried in partial sunlight. The weight of 50-seeds (SWt) of each fruit was weighed in grams by using the small digital weighing scale. Seed thickness (ST), seed length (SL), and seed width (SW) phenotypes were measured at their widest and longest axis, and the seed shape index (SSI) was calculated by dividing the SL over the SW (SL/SW). The seed coat of each seed was gently removed and the seed coat thickness (SCT) was also measured. The morphometric data of ST, SL, SW, and SCT was recorded in millimeter (mm) units by using a digital vernier caliper.

### Statistical data analysis

The multivariate phenotypic datasets were computed on Microsoft Excel (v2016) and statistical analysis were performed. The graphic illustration of analysis was done by using the R language tool (v3.2.3) coupled with the interface of RStudio (v1.0.143) (Wickham, 2009; R Core Team, 2018). The normality of the frequency distribution was checked based on the Shapiro-Wilk test at a significance *p*-value of <0.05. The representative qualitative and quantitative datasets with no missing values were used for the major variability by means of principal component analysis (PCA), respectively.

### Genomic sequencing and SNP mining

A sufficient amount (0.2 g) of juvenile leaves were sampled from the seedlings of comparative genotypes and high-quality GDNA was isolated by using the cetyl trimethyl ammonium bromide (CTAB) method (Allen et al., 2006). The density of isolated GDNA was checked on 1% agarose gel electrophoresis, and quickly stored at an ultra-low temperature (-80°C) before being used for molecular genotyping experiments. The quantified GDNA of both parent materials was fragmented by using the Diagenode Bioruptor Sonication device, and a sequencing library of 200–300 base pairs (bp) was pooled up by using the polymerase chain reaction (PCR) amplification (Amanullah et al., 2020). Whole genome sequencing of 10× genomic depth per sample was performed by using the High-throughput Illumina Sequencing™ 2000 technology at Beijing Genomics Institutes (BGI), Guangdong, P. R. China.

The quality of paired-end re-sequencing reads of comparative watermelon lines was checked and aligned over the downloaded *de novo* assembled genomic directory of watermelon (97103, v2 genome) using the perl script of Burrows-Wheeler Aligner (BWA) software package (v0.7.15-r1140) (Li and Durbin, 2009). Then, flexible generic sequence-alignment and map (SAM) text files and binary-alignment and map (BAM) index reads were sorted using the SAMtools program (v1.15.1) (http://www.htslib.org/). The whole genome filtered sequenced files were aligned and core sets of SNPs were mined using the bioinformatic tools “Genome Analysis Toolkit (GATK, v3.1-1) and VarScan (v2.0)” (McKenna et al., 2010; Ruffalo et al., 2011). SNPs were exported in variant call format (VCF) files by using the latest SnpEff (v5.1) (Cingolani et al., 2012) and annotated within intron and exon regions based on their genetic variant effects, respectively.

### CAPS markers validation

Whole genome CAPS markers were derived based on major SNP sequences, as reported in our earlier study (Amanullah et al., 2022). In brief, a total of 20–25 physical positions of SNP sequences “before and after 500 base pairs (bp)” of both parent lines were aligned across the whole gnome chromosomal length using SNP2CAPS software (Thiel et al., 2004). The suitable SNP sequences were chosen and converted into CAPS markers and DNA-based standard PCR primers were exported using the upgraded Primer Premier software (v6.25). The best quality primers were oligo-synthesized by Sangon Biotechology LTD., and then codominant polymorphic primers were screened based on distinct bands using the optimal CAPS PCR reactions in 10 μL mixture as previously stated (Amanullah et al., 2022), e.g., 0.5 μL of each primer sequence (forward + reverse), 1 μL PCR buffer, 0.1 μL *Taq* DNA-polymerase, 0.15 μL dNTPs, 1 μL GDNA, and 6.75 μL deionized water. The enzyme digested PCR products were cleaved on gel electrophoresis, and the digested bands were captured on the Invitrogen iBright Imaging Systems.

### QTL mapping and putative genes prediction

A watermelon linkage map was constructed and primary QTL mapping was done using QTL IciMapping software (Meng et al., 2015). In brief, the dataset of coded allelic bands of 100 F_2_ mapping population was recorded and arranged across the chromosomal length of watermelon genome. The recombination frequency of the linkage map was estimated among the adjacent markers based on genetic orders and intervals in centimorgans (cM). QTLs of ovary, fruit, and seed phenotypes were mapped on each chromosome, using the 1 cM sliding scale and 1000 permutation testing (*p* = 0.05). The threshold of a logarithm of odds (LOD) value of 2.5 and a genome-wide Type I (false-positive) error rate were used for checking the confidence intervals and genetic effects of identified QTLs between potential markers. The mapped QTLs with high LOD values (above 3) and phenotypic variance explained (PVE, >10%) were designated as major QTLs, and QTLs with low LODs and PVE (<10%) were designated as minor QTLs. Finally, the mapped QTLs were abbreviated as follows; the name of the trait, its position on the chromosome, and the number of QTLs, as reported earlier (Amanullah et al., 2022).

For the prediction of putative genes and their comprehensive genomic annotation, the physical sequences of adjacent QTL markers and comparative files of re-sequenced watermelon lines were pairwise aligned across the consensus directory of the watermelon genome (97103, v2), and genes numbers were visually tracked using the scalable visualization tool “Integrative Genomics Viewer (IGV, v2.12.2)” (Robinson et al., 2017). All the predicted genes were annotated based on the Gene Ontology (GO) function enrichments and Kyoto Encyclopedia of Genes and Genomes (KEGG) databases, as earlier described method (Yang et al., 2022).

## Results

### Analysis of WGS and SNPs identification

A total of 16.40 gigabytes (GB) of molecular data was obtained from whole-genome resequencing of two comparative parent lines and exposed 350 Mb and 354 Mb of genomic coverage across the watermelon genome directory (97103, v2), respectively. The mapping statistics of the WGS can be seen in **Supplementary Table S2**. Further, the detected SNPs and CAPS loci pairs were subsequently filtered through bio-informatics analysis, and the SNP distribution is shown in **Table 1**. A total of 352,177,534 bp of genomic length was effectively spanned across the whole-genome chromosomes (Chr-01 to Chr-11); among them, Chr-02 depicted the highest coverage with 36.80 Mb (36,805,829 bp) length, Chr-04 showed the minimum coverage of 26.10 Mb (26,100,705 bp), and other chromosomes demonstrated the differentiated coverage of genetic length (Mb) over the reference genome assembly.

**Table 1:** Distribution of SNPs and derived CAPS loci pairs across the total genetic length of watermelon genome.

Chromosomes	Genomic length	Total SNPs	SNPs rate/kb	CAPS loci
Chr-01	35,825,788	406,622	11	18,350
Chr-02	36,805,829	411,170	11	13,848
Chr-03	30,762,151	331,710	11	16,145
Chr-04	26,100,705	262,652	10	14,015
Chr-05	34,767,876	400,668	12	18,309
Chr-06	28,405,350	215,003	08	11,162
Chr-07	30,829,010	338,621	11	16,276
Chr-08	27,200,117	311,345	11	10,578
Chr-09	36,616,462	421,504	12	20,720
Chr-10	34,089,233	370,015	11	17,231
Chr-11	30,775,013	315,340	10	15,517
**Total**	**352,177,534**	**3,784,650**	**11**	**172,151**

The bold values denote the total calculation of each column data.

**Figure 2:**
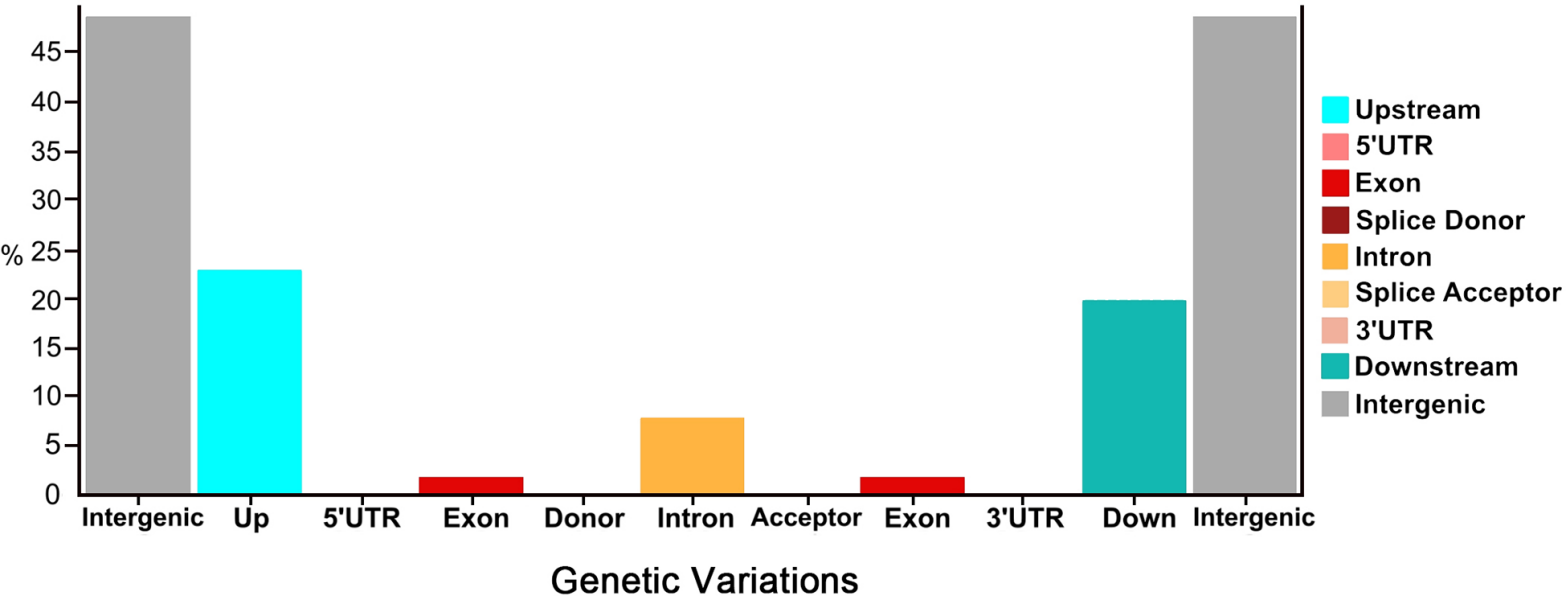
Proportions of genetic variations detected in the re-sequenced comparative parent lines of watermelon.

Overall, a total of 45.53% nsSNPs (missense) and 54.47% sSNPs (non-sense (53.94%) and silent mutations (0.53%)) were identified by their functional classes. These genetic variants were categorized into differential proportions with their number of produced effects by type and region (**Figure 2**). Regarding the genetic variants effects by type, the highest percentage was depicted in intergenic_region (48.44%), upstream_region (22.51%), and downstream_region (19.63%), while the minimum variants were exhibited in intron_region (7.75%) and exon_region (1.67%), followed by very less percentages of splice_site_acceptor, splice_site_donor, 5_prime_UTR_variant, and 3_prime_UTR_variant. Regarding the genetic variants effects by region, the highest percentage was depicted in intergenic_region (48.50%), upstream_region (22.54%), and downstream_region (19.65%), while minimum type of effects were present in intron_region (7.66%) and exon_region (1.65%), followed by very less percentages of splice_site_acceptor, splice_site_donor, 5_prime_UTR_variant, and 3_prime_UTR_variant, respectively.

### Analysis of CAPS markers

A total of 172,151 CAPS loci pairs were detected based on the appropriate cleaved sites of restriction endonucleases (RE) onto the whole-genome chromosomes. Overall, a good average of CAPSs was also observed across the remaining chromosomes; however, a maximum of 20,720 CAPSs were observed within the genetic length of Chr-09 and a minimum of 10,578 CAPSs were observed across Chr-08. A total of six different restriction enzymes (*Eco*R I, *Bsa*H I, *Msp* I, *Hin*d II, *Bam*H I, *Pst* I) were corresponded for cleaving the DNA within or adjacent to that site, and yielded the suitable pairs of CAPS markers sequences (*n*=454). The amplified PCR and digested products were assessed and a total of 210 sets of codominant adjacent CAPS markers were confirmed (**Supplementary Table S2**), by depicting 46.25% polymorphism among the different base pairs (bp) of P_1_, P_2_, and F_1_, respectively.

### Analysis of constructed linkage map

**Figure 3:**
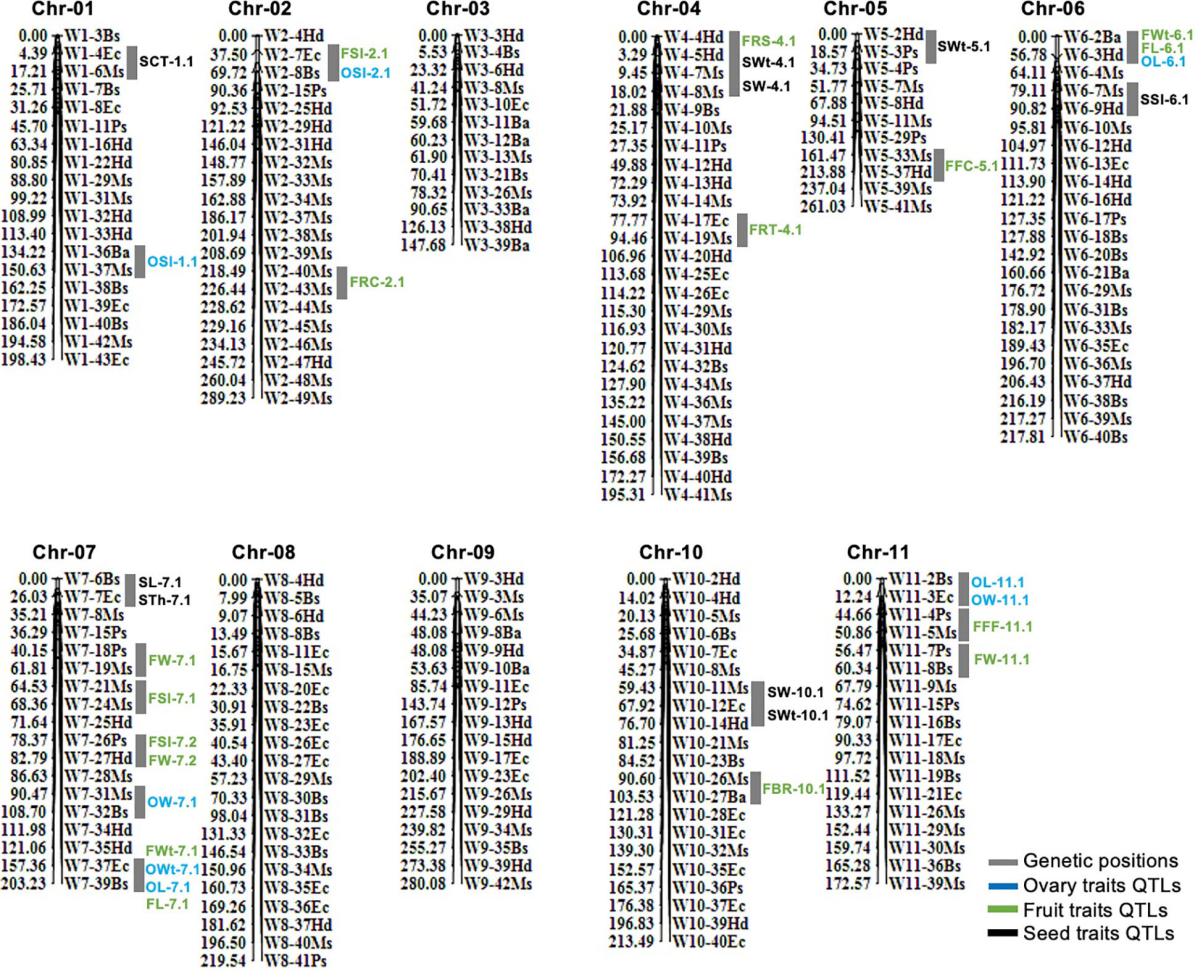
Genetic mapping and QTL analysis of ovary, fruit, and seed-related traits of watermelon. QTLs are mentioned in colored fonts and grey color chromosomal segments are indicating the genetic positions of QTLs.

In total, 210 pairs of codominant CAPS markers were genotyped within biparental F_2_ mapping families (*n*=100) and linkage map was constructed (**Figure 3**). A large portion of markers were localized over the developed linkage map (Chr-01 to Chr-11) according to their fragmented length (bp) and their physical positions in the sequences, and they showed perfect genotypic association and genome collinearity (*R* ^2^ = 0.924 to 0.995). A total of 195 markers (92.85%) displayed the perfect segregation ratio (*p*-value of >0.05) and just 15 markers (7.14%) exhibited biased segregation. The constructed linkage map enclosed a total length of 2,398.40 cM, having an average of 11.42 cM. However, whole-genome linkage group (LG) length varied from 147.68 cM (Chr-03) to 289.23 cM (Chr-02) length. The total number of codominant CAPS markers varied from a minimum of 11 (Chr-05 with 23.73 cM) to a maximum of 26 (Chr-04 with 195.31 cM length); however, an average of 10–15 markers were normally positioned on most of the chromosomes.

**Figure 4:**
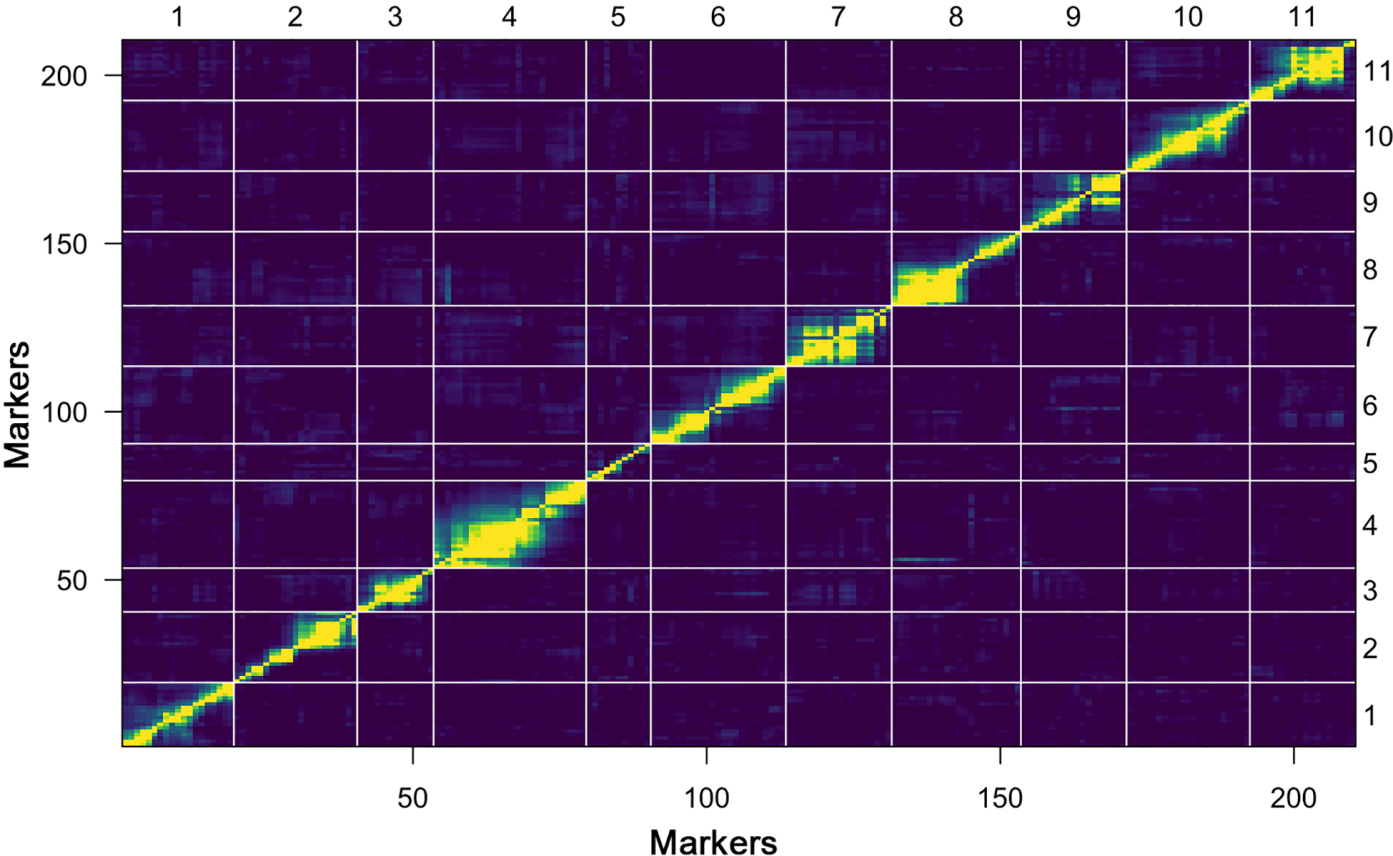
Plot of estimated pair-wise recombination fractions and LOD scores (upper-left and lower-right rectangles) of constructed genetic map.

Further, a heat map of 11 linkage groups (LGs) was generated based on pair-wise recombination values that illustrated the genomic collinearity of markers. The visualized linkage relations in the heat map exhibited the relationship between recombination of markers on each chromosome and this was used to identify the potential marker ordering. The order of markers on line and row was arranged according to their genetic distance. The closer the distance between different markers, the lower the recombination rate (indicated by the yellow color) was observed; however, a higher recombination rate is indicated by the purple color (**Figure 4**). The linkage rectangles (upper-left and lower-right) in the heat map generally indicate that the construction of our genetic map was accurate since the linkage groups were easily visualized.

### Phenotypic variation analysis

**Figure 5:**
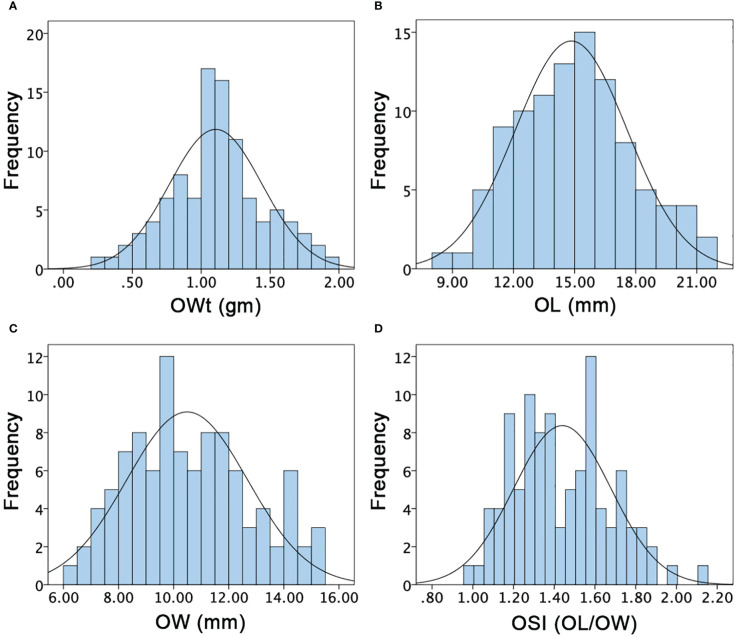
Histograms of the frequency distribution of ovary-related phenotypes in a developed F2:3 mapping population. **(A)** Ovary weight. **(B)** Ovary length. **(C)** Ovary width. **(D)** Ovary shape index.

The multivariate quantitative and qualitative datasets of ovary traits (OWt, OL, OW, OSI), fruit traits (FWt, FL, FW, FSI, FRS, FRC, FFC, FFF, FBR, FRT), and seed traits (SWt, SL, SW, SSI) were analyzed among the comparative watermelon lines (P_1_ and P_2_), F_1_ (off-spring), and F_2:3_ mapping families (**Figures 5** **-** **7**; **Supplementary Tables S4**-**S5**). A normal frequency distribution, strong transgressive segregation, significant correlation coefficients, and strong patterns of explained variability were observed among the phenotypic datasets, respectively.

### Analysis of ovary phenotypes

For the OWt, the mean values of parent lines (P_1_ and P_2_) were 0.73 ± 0.35 and 1.55 ± 0.30, and F_1_ offspring showed the different mean value of 0.88 ± 0.11, respectively. The overall OWt mean value of F_2:3_ mapping population was 1.10 ± 0.34 and major variability ranged from 0.30 to 1.90 gm (**Figure 5A**). For the OL, the mean values of P_1_, P_2_, and F_1_ were differentially noticed as 16.13 ± 1.22, 10.20 ± 0.35, and 12.43 ± 1.26, respectively. The overall OL mean of F_2:3_ mapping population was 14.83 ± 2.76 and variations ranged from 8.75 to 21.00 mm (**Figure 5B**). For the OW, the mean values of P_1_, P_2_, and F_1_ were also different (8.42 ± 0.34, 10.49 ± 0.11, and 7.45 ± 0.21), respectively. The overall OW mean values of F_2:3_ mapping population was 10.49 ± 2.19 and a varied range was seen with minimum of 6.29 to maximum of 15 mm (**Figure 5C**). The OSI of both parent lines and F_1_ was also dissimilar (1.92 ± 0.13, 1.05 ± 0.37, and 1.67 ± 0.12); however, the overall mean value of F_2:3_ mapping population was 1.44 ± 0.24 with a range of 0.98–2.14 (**Figure 5D**), respectively. Overall, the ovary associated phenotypes showed transgressive segregation and normal frequency distributions, indicating the inheritance of quantitative genetics with partial polygenic phenomena.

### Analysis of fruit phenotypes

**Figure 6:**
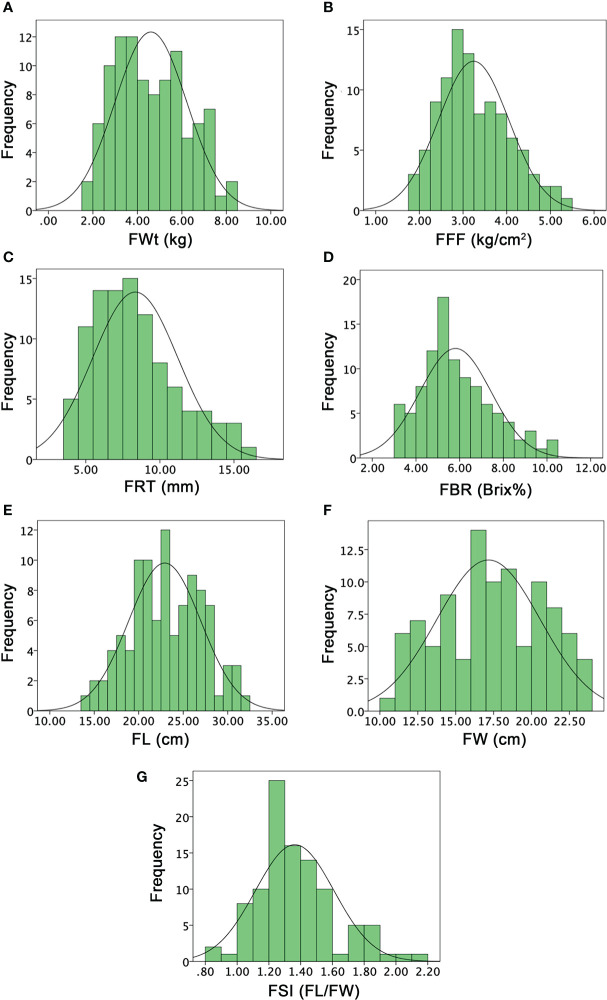
Histograms of the frequency distribution of fruit-related phenotypes in a developed F2:3 mapping population. **(A)** Fruit weight. **(B)** Fruit flesh firmness. **(C)** Fruit rind thickness. **(D)** Fruit Brix. **(E)** Fruit length. **(F)** Fruit width. **(G)** Fruit shape index.

The mean values of the FWT in parent lines (P_1_ and P_2_) were quite different (4.32 ± 0.14 and 5.73 ± 0.15), and their F_1_ offspring also showed more value (8.96 ± 0.11) than parent lines. The FWt mean value of F_2:3_ mapping population was 4.60 ± 1.62 and variations were ranged from 1.85 to 8.15 kg. A transgressive segregation and a normal frequency distribution were observed, and the major genetic variation effect in F_2:3_ fruit with medium to heavy weighted fruits was seemed to be inherited through F_1_ offspring (**Figure 6A**). FFF mean values of both parents were 1.29 ± 0.03 and 3.90 ± 0.13, and F_1_ offspring showed dissimilar mean value (3.37 ± 0.07). Among the F_2:3_ population, FFF mean value of fruit flesh rupturing force was ranged from 1.85 to 5.30 kg/cm^2^. A normal distribution frequency with lower to higher firmness values also illustrated the genetics of varied flesh texture (soft and hard) in fruits of F_2:3_ populations (**Figure 6B**). The flesh firmness variation was seemed as normally inherited by the genetic effects of wild male parent line with hard flesh firmness.

FRT was different in P_1_, P_2_, and F_1_ offspring (11.66 ± 0.58 and 8.16 ± 0.29, and 11.33 ± 0.58), respectively. However, the mean value of an F_2:3_ mapping population was noticed as 8.34 ± 2.88, with a varied range of 4 to 16 mm, exhibiting a normal quantitative frequency distribution (**Figure 6C**). Further, according to the visual observation of dissected fruits, most of the fruits of F_2:3_ mapping population showed more rind thickness and seemed that the cultivated female parent line (P_1_ with more rind thickness) and F_1_ depicted the major dominant effect of FRT inheritance; however, a few fruits showed less rind thickness. FBR was dissimilar in both parents (10.72 ± 0.20 and 3.80 ± 0.87) and their F_1_ offspring exhibited moderate sweetness level with mean value of 6.07 ± 0.11. The mean value of FBR of F_2:3_ population was noticed as 5.80 ± 1.63, with a varied range of 3 to 10%, respectively. The normal frequency distribution of Brix% explained a quantitative characteristic in F_2:3_ mapping population (**Figure 6D**), indicating the inherited nature of the female and sweet cultivated parent line (P_1_).

FL means of both parent lines were 26.17 ± 0.29 and 22.83 ± 0.76 and F_1_ offspring showed a different mean value of 33.83 ± 0.78. The overall FL mean value of F_2:3_ mapping population was 22.92 ± 4.07 and variations ranged from 14 to 31.50 cm, exhibiting the uniform frequency and transgressive segregation (**Figure 6E** **)**. FW means were also different in both parents (18.05 ± 0.51, 21.23 ± 0.75) and F_1_ (23.51 ± 0.50), respectively. However, the FW mean value was 17.16 ± 3.41 and the observed normal frequency distribution was ranged 10~23 cm, explaining the transgressive segregation in F_2:3_ population (**Figure 6F**). For both FL and FW traits, F_1_ fruits showed heterosis by describing the superior phenotypes relative to the parent lines and genetic effects of dominance were observed for obvious length and width in F_2:3_ family fruits, correspondingly. The FSI (FL/FW) of P_1_, P_2_, and F_1_ was manually deliberated and their mean values were 1.30 ± 0.38, 1.08 ± 0.36, 1.50 ± 0.25; however, the F_2:3_ mapping population mean value was 1.36 ± 0.25, having a range from 0.86 to 2.13, respectively (**Figure 6G**).

FRC of both parent lines exhibited different rind colors “dark-green (DG) and light green (LG)” and developed F_1_ offspring fruits showed an intermediate (I) color. The genetic inheritance of rind color in the F_2:3_ family fruits was based upon homozygous patterns in parent genotype of P_1_ (homozygous with dark green color), P_2_ (homozygous light green color), and F_1_ (heterozygous with intermediate color), e.g., 21 fruits with dark-green, 53 fruits with intermediate, and 26 fruits with light-green, exhibiting the 1:2:1 segregation ratio at *P*-value (0.12) and *χ* ^2^ value (4.39) (**Supplementary Table S5**). For the FFC, both parent lines illustrated dissimilar flesh colors “red (R), pale-green (PG)”, and their resultant F_1_ offspring showed a yellow (Y) color. In the developed F_2:3_ mapping population, most of the dissected fruits exhibited different and irregular flesh colors in the center part and placental tissues at the cross-sectional portion. So, the visible flesh color covering the maximum portion was considered as the dominant color and finally three colors were categorized, exhibiting the fitted genetic segregation of 1:2:1 at *P-*value (0.54) and *χ* ^2^ value (1.25), e.g., 21 fruits with red flesh, 56 fruits with yellow, and 23 fruits with pale-green flesh, proposing the major dominance of yellow flesh color (**Supplementary Table S5**). For the FRS appearance, fruits of P_1_ parent line exhibited wavy striped (W) and P_2_ parent line showed non-striped blotchy (B) type appearances; however, F_1_ fruits also showed wavy striped appearance but somewhat different from the female parent (P_1_). In the fruits of the developed F_2:3_ mapping population, genetic analysis showed that a total of 49 fruits were with with homozygous pattern of wavy stripes, 49 fruits were with wavy striped pattern related to F_1_ (striped but somewhat different from P_1_), and 30 fruits were with homozygous pattern of non-striped blotchy type, exhibiting the 1:2:1 segregation ratio (**Supplementary Table S5**). Thus, we assumed that the dominance effect of striped rind appearance on non-striped appearance was generally inherited and regulated by single locus. Overall, the analyzed datasets of FRC, FFC, and FRS suggested a largely but simply genetic inheritance pattern in the developed F_2:3_ mapping population.

### Analysis of seed phenotypes

**Figure 7:**
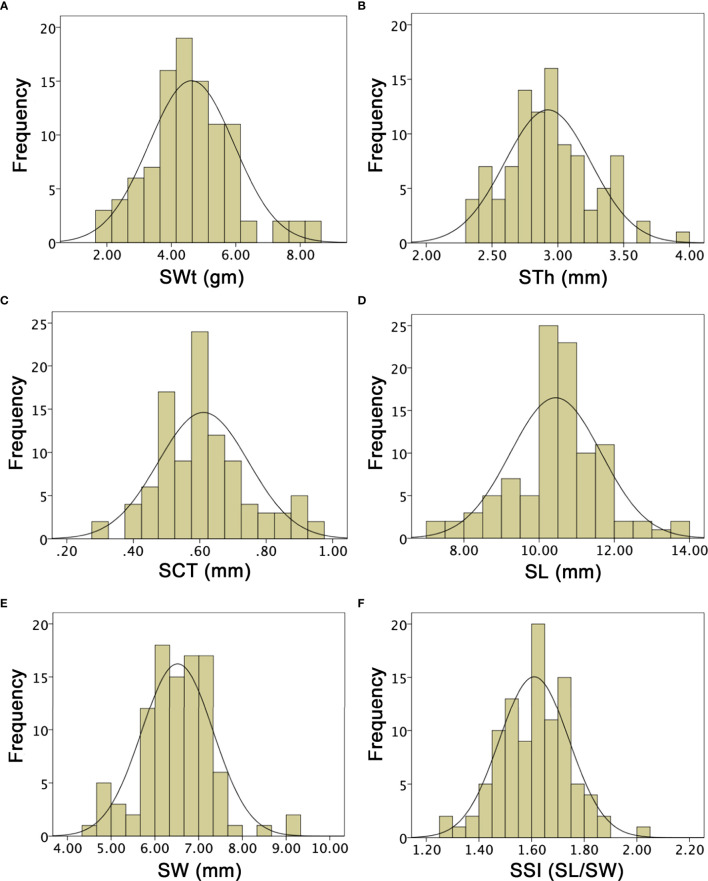
Histograms of the frequency distribution of seed-related phenotypes in a developed F2:3 mapping population. **(A)** Seed weight. **(B)** Seed thickness. **(C)** Seed coat thickness. **(D)** Seed length. **(E)** Seed width. **(F)** Seed shape index.

SWt of both parent lines and F_1_ were different and mean values were noticed as 4.20 ± 0.22, 5.40 ± 0.26, and 5.15 ± 0.23, based on their different genetic inheritance, respectively. In the developed F_2:3_ mapping population, the SWt mean was 4.62 ± 1.02 and ranged from 1.90 to 8.60 gm, by depicting a uniform quantitative distribution and transgressive segregation (**Figure 7A**). Overall, SWt genetic inheritance in mapping populations revealed close kinship with the wild type male parent line (P_2_). STh exhibited different mean values (1.89 ± 0.40, 2.73 ± 0.95, and 2.19 ± 0.16) in P_1_, P_2_, and F_1_, respectively. The STh mean value of F_2:3_ mapping population was 2.92 ± 0.33, and the ranged from 2.32 to 3.95 mm, indicating a uniform quantitative distribution and transgressive segregation (**Figure 7B**). Overall, genetic inheritance of STh in mapping populations showed an intimate relationship with the wild type male parent line (P_2_). SCT of both parent lines and F_1_ offspring had also distinct mean values (0.42 ± 0.03, 0.63 ± 0.02, and 0.53 ± 0.02), respectively. The SCT mean value of F_2:3_ mapping population was 0.61 ± 0.08, with a range of minimum 0.30 to maximum 0.95 mm. A uniform quantitative distribution and transgressive segregation was noticed (**Figure 7C**), disclosing the genetic effects of equal characteristics of parents and F_1_ offspring.

SL means of both parent lines were 9.64 ± 0.30 and 10.50 ± 0.75, and F_1_ had 8.65 ± 0.22. The overall SL mean value of F_2:3_ mapping population was 10.44 ± 1.21, a uniform distribution was observed, and variations ranged from 7.00 to 13.70 mm. SW means were also different as 18.05 ± 0.51, 21.23 ± 0.75, 23.51 ± 0.50, respectively. However, the SW mean value was 6.51 ± 0.82 and uniform frequency distribution was ranged 4.50 to 9.30 mm, that explained the transgressive segregation in F_2:3_ population (**Figures 7D, E**). It was found that obvious SL and SW characteristics of both parent lines were mutually shifted characteristics in developed F_1_ and F_2:3_ mapping populations. SSI (SL/SW) of P_1_, P_2_, and F_1_ was manually deliberated and their mean values were 1.67 ± 0.05, 1.57 ± 0.03, 1.62 ± 0.46; however, the F_2:3_ mapping population mean value was 1.61 ± 0.13, showing the range from 1.25 to 2.02 (**Figure 7F**), respectively.

**Figure 8:**
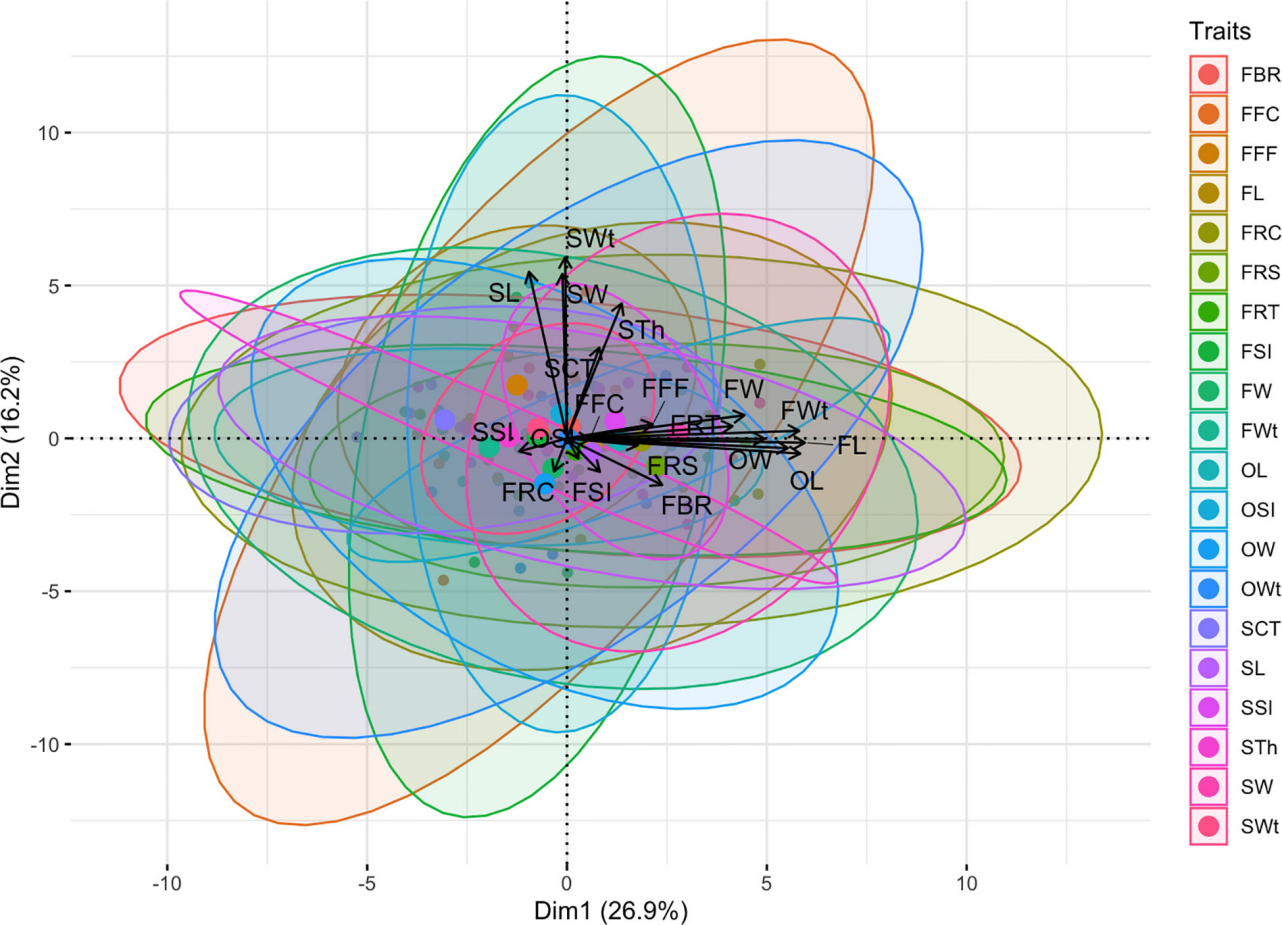
Principal component analysis (PCA) of multivariate phenotypes of watermelon.

In addition, the visualized biplot of principal component analysis (PCA) of multivariate phenotypic datasets explained a total of 43.10% shared major variability patterns and striking associations (**Figure 8**). The first dimension of principal component (Dim-1) extensively summarized the 26.90% explained variances among the seventeen phenotypic traits, and the second dimension of principal component (Dim-2) partially explained the 16.20% of three differentiated phenotypic traits. Overall, positive and linearly connected variables of ovary, fruit, and seed related phenotypes were seen at obtuse & acute angles, respectively.

### Analysis of QTLs/genes

A total of 33 QTLs (eight ovary QTLs, sixteen fruit QTLs, and nine seed QTLs) were classified that were randomly pinpointed on different genetic position among the whole-genome chromosomes (**Figure 3**); however, a QTL cluster was identified on Chr-07 and none of the QTL was observed on Chr-03 and Chr-09. Among the detected QTLs, a total of twenty four QTLs were identified as major-effect QTLs and nine QTLs were identified as minor-effect QTLs that explained the different LOD score values, PVE% (**Table 2**; **Supplementary Figure S1**), and significant SNP allelic effects for specific contributions (**Supplementary Figure S2**). The predicted genes and their detailed GO terms and KEGG pathway enrichment information are given in **Supplementary Tables S6**-**S8**, respectively.

**Table 2:** Genetic effects of mapped QTLs affecting multivariate phenotypes of watermelon.

QTLs	Chr.	Position (cM)	Adjacent markers	Position (bp)	LOD score	PVE (%)	Add effect	Dom effect
OWt-7.1	07	203	W7-37Ec~W7-39Bs	30359068~31916975	5.06	18.13	0.21	−0.05
OL-6.1	06	37	W6-2Ba~W6-3Hd	750440~1473640	4.22	25.99	−0.03	3.32
OL-7.1	07	203	W7-37Ec~W7-39Bs	30359068~31916975	3.60	8.91	1.48	−0.10
OL-11.1	11	0	W11-2Bs~W11-3Ec	793295~1543619	2.85	5.94	−0.46	−1.49
OW-7.1	07	101	W7-31Ms~W7-32Bs	25579724~26363747	3.52	14.06	−0.31	1.69
OW-11.1	11	10	W11-2Bs~W11-3Ec	793295~1543619	3.67	13.70	−0.01	−1.65
OSI-1.1	01	138	W1-36Ba~W1-37Ms	32266077~33187192	2.88	10.61	0.06	0.15
OSI-2.1	02	67	W2-7Ec~W2-8Bs	4733877~5684571	3.86	14.17	−0.12	−0.11
FRS-4.1	04	03	W4-4Hd~W4-5Hd	2031314~2731617	2.51	9.75	0.28	0.34
FRC-2.1	02	219	W2-40Ms~W2-43Ms	32909754~34162281	3.13	15.12	−0.28	−0.38
FWt-6.1	06	29	W6-2Ba~W6-3Hd	750440~1473640	3.87	15.61	−0.05	2.67
FWt-7.1	07	179	W7-37Ec~W7-39Bs	30359068~31916975	3.04	15.67	−0.16	2.65
FL-6.1	06	27	W6-2Ba~W6-3Hd	750440~1473640	3.16	20.48	−2.08	50.24
FL-7.1	07	203	W7-37Ec~W7-39Bs	30359068~31916975	2.60	5.32	19.34	1.95
FW-7.1	07	46	W7-18Ps~W7-19Ms	13585253~14389028	3.29	10.93	−10.96	20.93
FW-7.2	07	81	W7-26Ps~W7-27Hd	20772533~21571067	2.60	7.13	−9.68	15.46
FW-11.1	11	57	W11-7Ps~W11-8Bs	4623563~5404406	4.61	14.22	−22.61	−10.43
FSI-2.1	02	53	W2-7Ec~W2-8Bs	4733877~5684571	3.76	16.95	-0.15	-0.20
FSI-7.1	07	68	W7-21Ms~W7-24Ms	16745635~19169162	2.67	5.82	0.06	-0.13
FSI-7.2	07	79	W7-26Ps~W7-27Hd	20772533~21571067	3.04	6.42	0.06	-0.14
FFC-5.1	05	193	W5-33Ms~W5-37Hd	28704441~32310869	2.60	7.42	0.50	0.67
FFF-11.1	11	18	W114-Ps~W11-5Ms	2340516~3081651	2.57	9.01	−0.33	−0.13
FRT-4.1	04	78	W4-17Ec~W4-19Ms	10841468~12199409	3.02	14.48	−1.40	−0.69
FBR-10.1	10	91	W10-26Ms~W10-27Ba	21923221~22813961	2.94	13.98	0.08	1.22
SWt-4.1	04	7	W4-5Hd~W4-7Ms	2731617~4061374	2.91	13.85	−0.58	−2.00
SWt-5.1	05	15	W5-2Hd~W5-3Ps	886916~1795952	2.61	10.72	1.23	-0.02
SWt-10.1	10	69	W10-12Ec~W10-14Hd	9647097~11399426	2.89	11.54	−1.52	0.34
STh-7.1	07	14	W7-4Ba~W7-6Bs	3978006~4794459	2.51	11.87	−0.18	0.09
SL-7.1	07	7	W7-6Bs~W7-7Ec	3978006~4794459	6.77	23.82	−0.86	0.34
SW-4.1	04	13	W4-7Ms~W4-8Ms	4061374~4741494	2.79	13.78	−0.18	-0.59
SW-10.1	10	64	W10-11Ms~W10-12Ec	8770056~9647097	2.73	12.28	−0.52	0.30
SSI-6.1	06	83	W6-7Ms~W6-9Hd	4421727~5895809	2.76	13.41	0.07	−0.07
SCT-1.1	01	13	W1-4Ec~W1-6Ms	2773065~4611685	3.54	16.80	−0.07	0.07

### QTLs of ovary phenotypes

For the OWt, one major QTL (OWt-7.1) was detected at the bottom-end position on Chr-07. This QTL was also found to be closely related to the QTLs for fruit length and ovary length (FL-7.1 and OL-7.1), indicating a strong relationship. QTL of OWt-7.1 justified the individual genetic effect for ovary weight, with a total of 18.13% PVE, LOD score of 5.06, additive effect of 0.21, and dominance effect of −0.05. The genetic position of OWt-7.1 was spotted at 203 cM between the confidence interval of CAPS markers (W7-37Ec~W7-39Bs) situated at 157.36 cM and 203.23 cM and exhibited a genetic interval of 45.87 cM. However, the adjacent physical positions (30359068~31916975 bp) of markers exhibited a total of 1.56 Mb of interval that depicted a total of 168 putative genes.

For the OL, a total of three QTLs (one major QTL “OL-6.1” and two minor QTLs “OL-7.1, OL-11.1”) were differentially spotted at genetic positions of three distinct chromosomes (Chr-06, Chr-07, and Chr-11). On Chr-06, a major QTL (OL-6.1) was identified along with the fruit trait QTLs (FWt-6.1 and FL-6.1) and justified the individual genetic effect for ovary length, with a total of 25.99% PVE, LOD score of 4.22, negative additive effect of −0.03, and dominance effect of −3.32. The genetic position of OL-6.1 was spotted at 37 cM between the identified CAPS markers (W6-2Ba~W6-3Hd) positioned at 0.00 cM and 56.78 cM; however, the physical positions (750440~1473640 bp) of detected adjacent markers disclosed a total of 723.20 kb interval that depicted a total of 71 putative genes. On Chr-07, a minor QTL (OL-7.1) was tightly located with other QTLs (FWt-7.1, FL-7.1, OWt-7.1), and justified the individual trait effect with 8.91% PVE, LOD score of 3.60, positive additive effect of 1.48, and dominance effect of −0.10. The genetic position of OL-7.1 was spotted at 203 cM between the flanking markers (W7-37Ec~W7-39Bs) situated at 157.36 cM and 203.23 cM, which exhibited the genetic interval of 45.87 cM. However, the physical position (30359068~31916975 bp) of markers exhibited a total of 1.56 Mb interval that depicted a total of 168 putative genes. On Chr-11, a minor QTL (OL-11.1) was identified along with a single QTL of ovary width (OW-11.1), that explained the individual trait effect with 5.94% PVE, LOD score of 2.85, negative additive effect of −0.46, and dominance effect of −1.49. The low PVE% might be due to the quantitative nature of measured traits. The genetic position of OL-11.1 was spotted at the start of chromosomal segment at 203 cM between the flanking markers (W11-2Bs~W11-3Ec) situated at 0.00 cM and 12.24 cM, which spanned the moderate genetic interval of 12.24 cM. However, the physical position (793295~1543619 bp) of markers exhibited a total of 750.33 kb of interval that depicted a total of 73 putative genes.

For the OW, two major QTLs (OW-7.1 and OW-11.1) were identified on Chr-07 and Chr-11, and collectively explained 27.76% of the phenotypic variations. On Chr-07, OW-7.1 explained total 14.06% PVE, with LOD score of 3.52, negative additive effect of −0.31, and dominance effect of 1.69. The genetic position of OW-7.1 was marked at 101 cM between the flanking markers (W7-31Ms~W7-32Bs) situated at 90.47 cM and 108.70 cM, and spanned 18.23 cM interval. However, the physical position (25579724~26363747 bp) of pointed markers exhibited a total of 784.02 kb of interval that depicted a total of 61 putative genes. On Chr-11, OW-11.1 explained an individual effect of 13.70% PVE, with LOD score of 3.67, negative additive effect of −0.01, and dominance effect of -1.65. The genetic position of OW-11.1 was marked at 10 cM between the flanking markers (W11-2Bs~W11-3Ec) situated at 0.00 cM and 12.24 cM, which spanned a genetic interval of 12.24 cM. However, the physical position (793295~1543619 bp) of flanking markers exhibited a total of 750.33 kb of interval that depicted a total of 73 putative genes.

For the OSI, two major QTLs (OSI-1.1 and OSI-2.1) were detected on Chr-01 and Chr-02, and showed 25.32% phenotypic variation for shape indexes. On Chr-01, OSI-1.1 explained individual genetic effect of 10.61% PVE, with LOD score of 2.88, positive additive effect of 0.06, and dominance effect of 0.15. The genetic position of this QTL was situated at 138 cM between the flanking markers (W1-36Ba~W1-37Ms) situated at 134.22 cM and 150.63 cM, and spanned total 16.41 cM. However, the physical positions (32266077~33187192 bp) exhibited 921.12 kb interval that depicted a total of 134 putative genes. On Chr-02, another OSI-2.1 explained individual genetic effects of 14.17% PVE, with a LOD score of 3.86, negative additive effect of −0.12, and dominance effect of −0.11. The genetic position of this QTL was located at 67 cM between the flanking markers (W2-7Ec~W2-8Bs) situated at 37.50 cM and 69.72 cM, spanning 32.22 cM; however, the physical positions (4733877~5684571 bp) exhibited a 950.70 kb of interval that depicted a total of 73 putative genes. Regarding the overall inherited quantitative genetic of ovary related traits, the detected positive and negative additive effects of mapped QTLs exhibited multiple ovary characteristics (OWt, OL, OW, OSI) in a developed F_2:3_ mapping population and mainly signified the mutual heredity of both parent lines.

### QTLs of fruit phenotypes

For the FWt, two major QTLs (FWt-6.1 and FWt-7.1) were mapped on different chromosomes “Chr-06 and Chr-07” and collectively explained 31.28% of the phenotypic variance for fruit weight morphology. On Chr-06, FWt-6.1 was positioned at the start of the chromosomal segment along with fruit length and ovary length QTLs (FL-6.1 and OL-6.1), which explained phenotypic variance with 15.61% PVE, with LOD value of 3.87, negative additive effect of −0.05, and dominance effect of 2.67. The genetic position of this QTL was situated at 29 cM between the flanking markers (W6-2Ba~W6-3Hd) positioned at 0.00 cM and 56.78 cM, spanning a wide genetic distance of about 56.78 cM. However, the physical positions (750440~1473640 bp) exhibited a 723.20 kb interval that depicted a total of 71 putative genes. On Chr-07, FWt-7.1 was positioned at the bottom end of the chromosomal segment, along with the QTLs of ovary weight, ovary length, and fruit length (OWt-7.1, OL-7.1, FL-7.1), respectively. This QTL “FWt-7.1” explained phenotypic variance with 15.67% PVE, with LOD value of 3.04, negative additive effect of −0.16, and dominance effect of 2.65. The genetic position of this QTL was situated at 179 cM between the flanking markers (W7-37Ec~W7-39Bs) positioned at 157.36 cM and 203.23 cM, spanning a wide genetic distance about 45.87 cM, but adjacent physical positions (30359068~31916975 bp) exhibited a 1.56 Mb interval that showed a total 168 putative genes.

For the FL, one major QTL (FL-6.1) and one minor QTL (FL-7.1) were detected on genetic positions of different chromosomes (Chr-06 and Chr-07), and collectively explained 25.80% of the phenotypic variations for fruit length characteristics. On Chr-06, QTL “FL-6.1” was detected with LOD score of 3.16, negative additive effect of −2.08, dominance effect of 50.24, and explained individual phenotypic effect with 15.67% PVE. The genetic position of this QTL was situated at 27 cM between the flanking markers (W6-2Ba~W6-3Hd) positioned at 0.00 cM and 56.78 cM, spanning a wide genetic distance (cM). The adjacent physical positions (750440~1473640 bp) showed a 723.20 kb interval that depicted a total of 71 putative genes. On Chr-07, QTL “FL-7.1” was positioned at the end of corresponded chromosomal region and detected with LOD score of 2.60, positive additive effect of 19.34, dominance effect of 1.95, and explained individual trait effect with 15.67% PVE. The genetic position of this QTL was situated at 203 cM between the flanking markers (W7-37Ec~W7-39Bs) positioned at 157.36 cM and 203.23 cM, spanning a wide genetic distance of about 45.87 cM, but adjacent physical positions (30359068~31916975 bp) exhibited a 1.56 Mb interval that contained a total of 168 putative genes.

For the FW, a total of three QTLs (one minor QTL “FW-7.2” and two major QTLs “FW-7.1 and FW-11.1”) were detected on different genetic positions of Chr-07 and Chr-11, and collectively explained 32.28% of the phenotypic variances for fruit width descriptions. On Chr-07, a major QTL (FW-7.1) was found with LOD score of 3.29, negative additive effect of −10.96, dominance effect of 20.93, and a 10.93% PVE, individually. The genetic position of this QTL was located at 46 cM between the flanking markers (W7-18Ps~W7-19Ms) positioned at 40.15 cM and 61.81 cM, spanning total 21.66 cM genomic interval, but adjacent physical positions (13585253~14389028 bp) showed 803.78 kb interval, displaying a total 7 putative genes. Further, a minor QTL (FW-7.2) was noticed with LOD score of 2.60, negative additive effect of −9.68, dominance effect of 15.46, and a 7.13% PVE, individually. The genetic position of this QTL was located at 81 cM between the flanking markers (W7-26Ps~W7-27Hd) positioned at 78.37 cM and 82.79 cM, spanning delimited genomic interval of 4.42 cM, but adjacent physical positions (20772533~21571067 bp) showed a 798.54 kb interval, showing a total of 35 putative genes. On Chr-11, a single major QTL (FW-11.1) was identified with LOD score of 4.61, negative additive effect of −22.61, and dominance effect of −10.43 and individually depicted 14.22% PVE. The genetic position of this QTL was situated at 57 cM between the flanking markers (W11-7Ps~W11-8Bs) positioned at 56.47 cM and 60.34 cM, spanning a shortened genomic interval (3.87 cM), but adjacent physical positions (4623563~5404406 bp) exhibited a 780.85 kb interval, disclosing a total 88 putative genes.

For the FSI, a total of three QTLs “one major QTL (FSI-2.1) and two minor QTLs (FSI-7.1 and FSI-7.2)” were localized on the genetic positions of two differential chromosomes (Chr-02 and Chr-07). On Chr-02, FSI-2.1 QTL was positioned along with OSI-2.1 and exhibited a good genetic connection between ovary and fruit shape. This QTL explained individual genetic effects of 16.95% PVE, with LOD score of 3.76, negative additive effect of −0.15, and dominance effect of −0.20. The genetic position of FSI-2.1 was found at 53 cM between the flanking markers (W2-7Ec~W2-8Bs) situated at 37.50 cM and 69.72 cM, that spanned 32.22 cM; however, the physical position (4733877~5684571 bp) exhibited 950.70 kb interval that depicted a total 73 putative genes. On Chr-07, FSI-7.1 QTL was located separately but near to the genetic position of FW-7.1 and explained the individual effect with 5.82% PVE, positive additive effect of 0.06, and negative dominance effect of −0.06. The genetic position of FSI-7.1 was found at 68 cM between the flanking markers (W7-21Ms~W7-24Ms) situated at 64.53 cM and 68.36 cM, spanning a shortened genetic interval of 3.83 cM; however, the physical position (16745635~19169162 bp) exhibited a 2.20 Mb interval, enclosing a total of 21 putative genes. Another QTL (FSI-7.2) seemed as tightly localized as the FW-7.2 QTL, perhaps signifying that fruit shape was mainly determined by fruit width. The genetic position of FSI-7.2 was situated at 79 cM between the flanking markers (W7-26Ps~W7-27Hd) positioned at 78.37 cM and 82.79 cM, and spanned a shortened genetic interval of 4.42 cM. The physical position (20772533~21571067 bp) exhibited a 798.54 kb interval, revealing a total of 35 putative genes.

For the FRT, one major QTL (FRT-4.1) was mapped at Chr-04 and explained an individual genetic effect with 14.48% PVE, LOD score of 3.02, negative additive and dominance effects (−1.40 and −0.69). The genetic position of this QTL was situated in the middle genetic section of Chr-04, at 78 cM between the flanking markers (W4-17Ec~W4-19Ms) positioned at 77.47 cM and 94.46 cM, spanning a total genetic interval of 16.69 cM. However, the physical position (10841468~12199409 bp) exhibited a 1.36 Mb interval that showed a total of 18 genes. For the FFF trait, one minor QTL (FFF-11.1) was mapped on Chr-11 and this QTL explained an individual genetic effect with 9.01% PVE, LOD score of 2.57, negative additive and dominance effect (−0.33 and −0.13). The genetic position of this QTL was situated at 18 cM between the flanking markers (W11-4Ps~W11-5Ms) positioned at 44.66 cM and 50.86 cM, covering a minimum of 6.20 cM, and the physical position (2340516~3081651 bp) exhibited total 741.14 kb interval, depicting 78 putative genes. For the FBR trait, one major QTL (FBR-10.1) was mapped at Chr-10 and explained individual phenotypic effects with 13.98% PVE, LOD score of 2.94, and positive additive and dominance effects of 0.08 and 1.22. The genetic position of this QTL was situated at 91 cM between the flanking markers (W10-26Ms~W10-27Ba) positioned at 90.60 cM and 103.53 cM, spanning a genetic interval of 12.93 cM. The physical position (21923221~22813961 bp) exhibited an 890.74 kb interval that showed a total of 43 genes.

For the FFC, one minor QTL (FFC-5.1) was mapped to a wide chromosomal region of Chr-02 and explained individual genetic effects with 7.42% PVE, LOD score of 2.60, positive additive and dominance effects (0.50 and 0.67). The genetic position of this QTL was situated at 193 cM between the flanking markers (W5-33Ms~W5-37Hd) positioned at 161.47 cM and 213.88 cM, spanning a broad range of genetic interval (52.41 cM); however, the physical positions (28704441~32310869 bp) exhibited a 3.61 Mb interval that depicted a total of 475 genes. For the FRC trait, one major QTL (FRC-2.1) was mapped at the bottom end of Chr-02. This QTL explained individual genetic effects of 15.12% PVE, with LOD score of 3.13, negative additive effect of −0.28, and dominance effect of −0.38. The genetic position of this QTL was situated at 219 cM between the flanking markers (W2-40Ms~W2-43Ms) positioned at 218.49 cM and 226.44 cM, spanning just 9.80 cM. However, the physical position (32909754~34162281 bp) exhibited a 1.25 Mb interval that depicted a total of 157 putative genes. For the FRS trait, one minor QTL (FRS-4.1) was identified at the start position of Chr-04. This QTL explained individual genetic effects of 9.75% PVE, with LOD score of 2.51, positive additive effect of 0.28, and dominance effect of 0.34. The genetic position of this QTL was situated at 3 cM between the flanking markers (W4-4Hd~W4-5Hd) positioned at 0.00 cM and 3.29 cM, spanning a total of 3.29 cM. However, the physical positions (2031314~2731617 bp) exhibited a 700.30 kb interval and depicted a total of 16 putative genes.

### QTLs of seed phenotypes

For the SWt, a total of three major QTLs (SWt-4.1, SWt-5.1, and SWt-10.1) were detected on three different chromosomes (Chr-04, Chr-05, and Chr-10). Interestingly, these QTLs shared a strong relationship with seed width and seed length QTLs (SW-4.1 and SW-10.1), indicating that seed weight was primarily inherited by seed width characteristics. On Chr-04, SWt-4.1 QTL was mapped with LOD score of 2.91, negative additive and dominance effects (−0.58 and −2.00), and mainly explained the phenotypic variation for reduced seed weight with 13.85% PVE. The genetic position of SWt-4.1 QTL was situated at 7 cM between the flanking markers (W4-5Hd~W4-7Ms) placed at 3.29 cM and 9.45 cM, covering 6.16 cM; however, the physical position (4061374~4741494 bp) unveiled a total 680.12 kb interval that depicted 32 putative genes. On Chr-05, SWt-5.1 QTL was pinpointed with LOD score of 2.61, positive additive effect of 1.23, and a negative dominance effect of −0.02, and mainly explained the phenotypic variation for more seed weight with 10.72% PVE. The genetic position of SWt-5.1 QTL was situated at 15 cM between the flanking markers (W5-2Hd~W5-3Ps) sited at 0.00 cM and 18.57 cM; however, the physical position (886916~1795952 bp) unveiled a total 909.04 kb interval predicted for 135 genes. On Chr-10, SWt-10.1 QTL was pinpointed with LOD score of 2.89, negative additive effect of −1.52 and a positive dominance effect of 0.34, and explained the phenotypic variation for less seed weight with 11.54% PVE. The genetic position of SWt-10.1 QTL was situated at 69 cM between the flanking markers (W10-12Ec~W10-14Hd) situated at 67.92 cM and 76.70 cM, and the physical positions (9647097~11399426 bp) revealed a 1.75 Mb interval having 51 genes.

For the STh and SL, two major QTLs (STh-7.1 and SL-7.1) were pinpointed on Chr-07. They shared a common genetic location by explaining the combined characteristics of reduced seed thickness as well as seed length and seemed to determine the genetic inheritance by somewhat moderate-sized seeds having less seed thickness, respectively. For the STh, one major QTL (STh-7.1) was pinpointed with LOD score of 2.51, negative additive effect of −0.18, positive additive effect of 0.09, and 11.87% PVE. For the SL, a major QTL (SL-7.1) was detected with LOD score of 6.77, negative additive effect of −0.86, positive dominance effect of 0.34, and 23.82% PVE, separately. The genetic positions of both QTLs were found at 14 cM and 7 cM between the adjacent flanking markers (W7-6Bs~W7-7Ec) situated at 0.00 cM and 26.03 cM, and their physical positions (3978006~4794459 bp) exhibited an interval of 1.59 Mb, which contained 46 genes.

For the SW, two major QTLs (SW-4.1 and SW-10.1) were found on two different chromosomes (Chr-04 and Chr-10). These two QTLs were found to be close to the seed weight QTLs on those chromosomes, showing that seed weight and seed width are closely related. On Chr-04, a major QTL (SW-4.1) justified the individual genetic effect for seed weight, with a total of 13.78% PVE, LOD score of 2.79, negative additive and dominance effects of −0.18 and −0.59. The genetic position of SW-4.1 was spotted at 13 cM between the flanking sections of two CAPS markers (W4-7Ms~W4-8Ms) situated at 9.45 cM and 18.02 cM, and exhibited the genetic interval of 8.57 cM. But the physical positions of markers next to each other (4061374–4741494 bp) only showed 8 putative genes over a total of 680.12 kb. On Chr-10, another major QTL (SW-10.1) explained the genetic effects with 12.28% PVE, LOD score of 2.73, negative additive effect of −0.52, and dominance effect of 0.30. The genetic location of SW-10.1 was found to be 64 cM between the CAPS markers W10-11Ms and W10-12Ec, which were at 59.43 cM and 67.92 cM, with a genetic gap of 8.49 cM. However, the adjacent physical positions (8770056~9647097 bp) of markers exhibited a total of 877.04 kb of interval that depicted just 36 putative genes.

For the SSI, one major QTL (SSI-6.1) was mapped over Chr-06 and explained individual genetic effects with 13.41% PVE, with LOD score of 2.76, positive additive effect of 0.07, and negative dominance effect of −0.07. The genetic position of this QTL was situated at 83 cM between the flanking markers (W6-7Ms~W6-9Hd) positioned at 79.11 cM and 90.82 cM, spanning about 11.71 cM; however, the adjacent physical positions (4421727~5895809 bp) exhibited a total 1.47 Mb interval and showed 146 putative genes. For the SCT trait, one major QTL (SCT-1.1) was separately mapped to the chromosomal region of Chr-01 and explained individual genetic effects with 16.80% PVE, with LOD score of 3.54, negative additive effect of −0.07, and positive dominance effect of 0.07. The genetic position of this QTL was situated at 13 cM between the flanking markers (W1-4Ec~W1-6Ms) situated at 4.39 cM and 17.21 cM, covering 12.82 cM; however, the adjacent physical positions (2773065~4611685 bp) exhibited total 1.84 Mb interval and displayed a total of 187 putative genes.

### GO and KEGG enrichment analysis of predicted genes

In total, 580 genes for four ovary traits, 1172 genes for ten fruit phenotypes, and 641 genes for six seed traits were identified among the identified flanking QTL regions (**Supplementary Table S6**), and SNP allelic effects underlying identified QTLs also shown the significant allele specific contributions (**Supplementary Figure S2** **)**, respectively.

According to the pair-wise sequence analysis of parent line sequences and reference genome assembly, most of the genes showed candidate mutations within the CDS coding regions. Moreover, the genes were subsequently predicted and analyzed to check their categorized synteny of potential mechanisms mediating the ovary, fruit, and seed traits of comparative experimental material.

**Figure 9:**
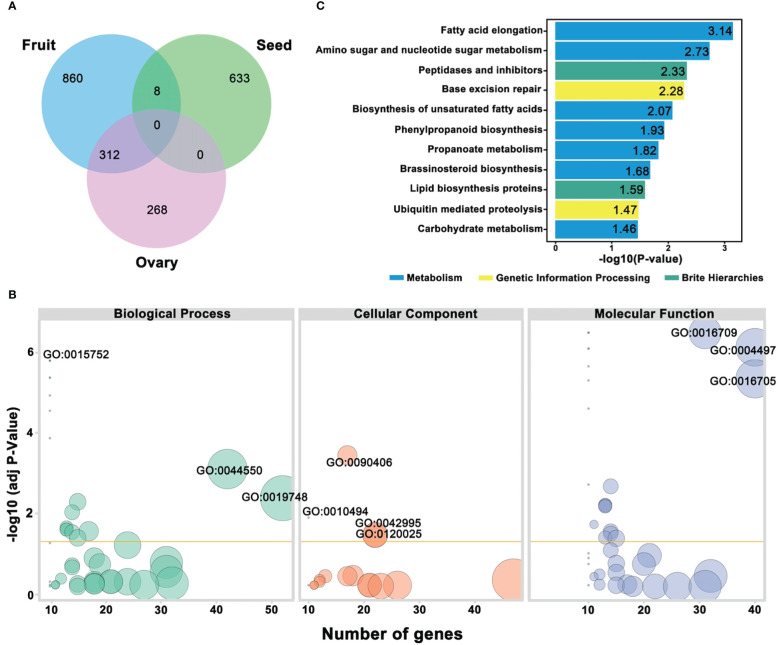
Annotation analysis of predicted genes underlying mapped genomic regions **(A)** Venn diagram of categorized genes. **(B)** GO functional enrichment analysis. **(C)** KEGG pathway enrichment analysis, respectively.

A schematic representation of the VENN diagram significantly illustrated the categorized genes (**Figure 9A**), and a total of 312 genes were observed to show the decisive functions for regulating the dynamic traits of ovary and fruit, and only 8 genes exhibited a connection between fruit and seed traits, respectively. Gene Ontology function enrichment analysis exhibited the functional distribution of ovary, fruit, and seed traits linked genes (**Supplementary Table S7**). According to the bio-informatic analysis, GO functional enrichment was mainly categorized into molecular function (MF), biological process (BP), and cellular components (CC), and GO terms with a mean *P*-value of <0.05 were recognized as considerably enriched (**Figure 9B**).

Regarding the GO biological process, the identified genes were divided into 19 significant GO terms with different values of -log10(*P*-value); among them, GO:0015752 (D-ribose transmembrane transport), GO:0044550 (secondary metabolite biosynthetic process), and GO:0019748 (secondary metabolic process) were found with highly enriched GO terms. The low numbers of GO terms were cellular response to metal ion, glucose transmembrane transport, hexose transmembrane transport, and glucose import. However, few other genes were found with a moderate number of GO terms. In the GO cellular component, 4 GO terms were exhibited as highly enriched, e.g., GO:0090406 (pollen tube) GO:0010494 (cytoplasmic stress granule), GO:0042995 (cell projection), and GO:0120025 (plasma membrane bounded cell projection), and the remaining were detected with low to moderate enrichment. All the genes were divided into 24 significant GO terms in the GO molecular function. Three of these terms were significantly enriched; GO:0016709 (oxidoreductase activity acting on paired donors with incorporation or reduction of molecular oxygen of NAD(P)H as one donor, and incorporation of one atom of oxygen), GO:0004497 (D-xylose transmembrane transporter activity), and GO:0016705 (oxidoreductase activity, acting on paired donors, with incorporation or reduction of molecular oxygen). The GO terms with the lowest significant enrichment were hexose transmembrane transporter activity, glucose transmembrane transporter activity, phosphatidic acid binding, carbohydrate: proton symporter activity, carbohydrate: cation symporter activity, oxidoreductase activity, hydro-lyase activity, poly(A) binding, solute: cation symporter activity, and symporter activity; however, the remaining GO terms were noticed with moderate enrichment.

To better understand the important gene contributions in the key metabolic and signal transduction pathways regulating the ovary, fruit, and seed phenotypes of comparative parental lines, the identified genes were evaluated in the KEGG database, and the first 11 pathways with 3 main classes were designated at significant -log10(*P*-value) and used for visualized plotting (**Figure 9C**; **Supplementary Table S8**). The most significantly enriched pathways were observed with fatty acid elongation (Ko00062), amino sugar and nucleotide sugar metabolism (Ko00520), peptidase and inhibitors (Ko01002), lipid biosynthesis proteins (Ko01004), followed by Ko03410 (Base excision repair) and Ubiquitin mediated proteolysis, and the main classes were metabolism, genetic information processing, and brite hierarchies, which might be involved in the differentiation of the horticultural phenotypes of comparative parental lines, respectively.

## Discussion

Watermelon is an important fruit in the cucurbitaceae family with a wide range of quantitative and qualitative characteristics. In this experiment, two extremely divergent watermelon parent lines (W1-38 and PI542119) were used for genomic sequencing and a total of 3,784,650 SNPs and 172,151 CAPS loci pairs were detected; however, transition type SNPs were noticed higher than transversion type SNPs. A total of 210 sets of novel SNP-CAPS markers exhibited a moderate level of polymorphism (46.25%), and the obtained results are fairly comparable with the earlier reported genetic mapping studies in watermelon (Liu et al., 2015; Liu et al., 2016; Amanullah et al., 2021; Osae et al., 2021; Amanullah et al., 2022; Osae et al., 2022). Our constructed genetic map had a total length of 2,398.40 cM, an average interval length of 11.42 cM, and most physical intervals ranging from a minimum of 680.12 kb to a maximum of 950.70 kb, indicating lower recombination rate (**Figures 3**, **4**). Furthermore, a few CAPS markers displayed a relatively large genetic interval ranging from 1.5 Mb to 3.3 Mb and appeared to be deviated in the linkage equilibrium across the reference genome. The density of whole-genome markers with fewer genetic distances and the size of mapping populations are major concerns for perfect linkage mapping and QTL analysis (Pereira et al., 2018; Liang et al., 2022). So, we assumed that our developed genetic map still needs to be improved by delimiting the unsuitable genetic intervals by incorporating the high density markers that would provide the additional accuracy for QTLs/genes mapping.

### Ovary QTLs

A few QTLs of ovary-related specific traits (weight, width, length, and shape index) have been successfully analyzed in a few Cucurbitaceae fruits, including cucumber (Wei et al., 2016; Wang et al., 2020), melon (Ramamurthy and Waters, 2015; Amanullah et al., 2020; Amanullah et al., 2021), and squash (Kamiska et al., 2018). In watermelon, it was reported that development of ovary shape/size is a pre-anthesis genetic phenomenon developed at the initial development stage of the ovary, which similarly leads to the different obvious fruit shapes induced by polygenic control (Dou et al., 2018b). Due to pre-anthesis genetic inheritance in cultivars with different genetic backgrounds (Legendre et al., 2020), the long ovaries cause the relative absolute shape of the fruit to be long, and the round ovaries cause the fruit to be round.

For the genetic mapping of watermelon ovary traits, a molecular mapping study classified two ovary weight QTLs, two ovary length QTLs, and three ovary width QTLs, which were positioned on Chr-01, Chr-03, Chr-08, and Chr-09 (Osae et al., 2022) and explained about 8.87% to 20.82% PVE, by defining the polygenic architecture and conferring the obvious ovary-fruit shape index. In our study, ovary traits related to major and minor QTLs were mapped between the delimited adjacent regions of genetic markers positioned on Chr-01, Chr-02, Chr-06, Chr-07, and Chr-11, which explained 8.91% to 25.99% PVE, respectively (**Figure 3**; **Table 2**). The detected positive and negative additive effects of our mapped QTLs exhibited the variation of multiple ovary characteristics (OWt, OL, OW, and OSI) in the developed F_2:3_ mapping population and mainly signified the mutual genetic heredity of both parent lines. We also discovered that our mapped QTLs regions contradicted those previously mapped QTLs (Dou et al., 2018b; Legendre et al., 2020; Osae et al., 2022), as shown in **Supplementary Table S9**, respectively.

To the best of our knowledge, our identified QTL segments indicated the new genetic regions with strong pleiotropic effects for controlling the ovary traits. Furthermore, transgressive segregation was observed for all ovary traits in our study (**Figure 5**), and co-QTLs on Chr-02, Chr-06, and Chr-11 strongly supported the existence of synteny modulating between genetic positions in the watermelon genome. It is supposed that allelic fashions of both parent lines produced the genetic effects for reduced size and lengthy ovaries variation. Interestingly, the detected QTLs were also fitted with quantitative genetics of ovary associated traits and their mechanisms was seemed to be triggered by numerous genes.

### Fruit QTLs

Fruit weight (FWt) is very important for making a good commercial profit (Amanullah et al., 2021). The morphological divergences of different cultivars range from fruit weight in terms of gm to kg (Osae et al., 2022; Liang et al., 2022). In few earlier studies, QTLs of watermelon FWt have been mapped over Chr-09 and Chr-03 by using the developed biparental and RIL mapping families resulting from the crossing of cultivated-type and wild-type parent lines (Fan et al., 2000; Sandlin et al., 2012; Yang et al., 2021; Liang et al., 2022; Osae et al., 2022). In this study, we similarly incorporated biparental F_2:3_ mapping population and mapped just two major QTLs of fruit weight (FWt-6.1 and FWt-7.1) positioned over Chr-06 and Chr-07, which justified 15.61~15.67% PVE for FWt variation (less and more), respectively (**Figure 3**; **Table 2**). These QTL results contradict the earlier published studies (**Supplementary Table S9**) and strongly suggest the genetic divergence in contrast to the parental lines and their derived experimental populations. So, we hypothesized that our new FWt QTLs might signify the new reliable mapped genomic regions for controlling the variation in FWt of watermelon. For the genetic regulation of FWt mechanisms, the *LC* (an important member of the *WOX*, *YABBY*, and *FAS* families) was significantly known for gradual variation in FWt (Huang et al., 2013), and the *FW2.2/CNR* was similarly classified as a major locus of FWt that encodes the protein for relative regulation of cell number regulators (CNR) (Wu et al., 2018). Our identified genes for FWt might exhibit the gene expression profiling but strong validation is necessary by further fine mapping study.

Fruit size (length, width, and shape) variations in watermelon have been classified into elongated, round, blocky, or oval shapes (McKay, 1936; Wehner et al., 2001). Many studies have identified QTLs that control the majority of fruit variation in various biparental mapping populations, RILs, and natural populations under various environmental conditions (Lu et al., 2009; Sandlin et al., 2012; Ren et al., 2014; Liu et al., 2014; Kim et al., 2015; Liu et al., 2015; Liu et al., 2016; Cheng et al., 2016; Lu et al., 2016; Dou et al., 2018b; Maragal et al., 2019; Legendre et al., 2020). The inclusive number of QTLs regulating the watermelon shape/size have been reviewed across all previously published QTL results (Pan et al., 2020), and 9 inclusive QTLs have been reported across 7 distinct chromosomal regions of Chr-02, Chr-03, Chr-05, Chr-07, Chr-08, Chr-09, and Chr-10. The FSI QTL “ClFSI-3.1” was discovered on Chr-03, exhibiting stable genetic effects for the regulation of FSI in segregating biparental populations of watermelon. However, four QTLs (ClFS-2.3, ClFS-3.3, ClFS-4.1, and ClFS-8.1) could express stable interactions in more than one experimental mapping population or environmental location of the 15 inclusive FSI QTL (Sandlin et al., 2012). In this study, we pinpointed a total of eight QTLs with multiple-effects for fruit length, fruit width, and fruit shape index across the genomic intervals positioned on four different chromosomes (Chr-02, Chr-06, Chr07, and Chr-11) (**Figure 3**; **Table 2**). Two QTLs of fruit length (FL-6.1 and FL-7.1) were expressed as major-effect and minor-effect QTLs, which explained the fruit length variations (short size and large size fruits) with 20.48% and 5.82% PVE, respectively. Three QTLs of fruit width were identified (FW-7.1 “major-effect”, FW-7.2 “minor-effect”, and FW-11.1 “major-effect”), and contributed to the fruit width variations with 7.13% to 14.22% PVE. Three QTLs of fruit shape variations were further classified (FSI-2.1 “major-effect”, FSI-7.1 “minor-effect”, and FSI-7.2 “minor-effect”), and these QTLs contributed to most of the fruit width variations with 5.82~16.95% PVE. We noticed that our identified QTLs and their genetic positions are inconsistent with the previously published results, as shown in **Supplementary Table S9**, respectively.

Regarding the genomic co-linearity, the fruit size/shape related QTLs were noticed as tightly co-localized with ovary size/shape related QTLs, respectively. The co-localized QTLs (OL-6.1 and FL-6.1, OL-7.1 and FL-7.1, OSI-2.1, and FSI-2.1) significantly demonstrated that obvious shapes of long and wide fruits have high connectivity since the cell structure development at the ovary establishment stage. We also noticed that oblong shaped fruits of the F_2:3_ population have a genetic resemblance with the female watermelon parent (P_1,_ with an oblong shape) and moderate and rounded fruits have a resemblance with the male parent (P_2,_ rounded shape). Our identified fruit size/shape QTL results similarly suggested an inherited quantitative genetics and transgressive segregation (**Figure 6**), which is supposed to be controlled by polygenic architecture and is mainly regulated by dominant allelic fashions of comparative watermelon lines that primarily triggers the clear fruit shape variations of oblong and rounded fruit growth throughout the dynamic growth stages in fruits of F_2:3_ families.

Similarly, in melon, a pre-anthesis genetic structure explains the prominent polygenic regulatory mechanism (Ramamurthy and Waters, 2015; Amanullah et al., 2021). In cucumber (*Cucumis sativus*), a strong association was reported between the establishment of an ovary and fruit shape (Weng et al., 2015; Wei et al., 2016). However, the fruit size/shape is mainly determined by cell division and cell expansion during the vegetative and reproductive growth stages. The fruit shape index is triggered by a predominantly genetic mechanism in tomato, which has been identified at various developmental stages (Lippman and Tanksley, 2001; Eduardo et al., 2007; van der Graaff et al., 2009, van der Knaap et al., 2014; van der Knaap and Ostergaard, 2018). The near isogenic line (NILs) of tomato with the allelic nature of lengthy ovaries produced more elongated fruits than small-shaped NILs (Frary et al., 2000), and the modeling of FSI at flowering stage suggested a pleiotropic effect with a major drag effect of QTL and appeared to be handled by the OVATE gene family (Ku et al., 2000). Actually, fruit length and width increment is not a continual development near the proximal distal axis; but it depends upon the gradual cell division process that occurs during the ovary formation (van der Knaap and Ostergaard, 2018).

Overall, our identified results are in accordance with the few earlier published studies; e.g., it has been stated that differences concerning the elongate/oblong and rounded watermelon shapes can be identified by identical ovaries at pre-anthesis stages, and a single gene with incomplete dominance locus “*O*”, differential genotypes “*OO*, *oo*, *Oo*,” are mainly responsible for the obvious shapes of elongated, rounded, and blocky shapes (Weetman, 1937; Poole and Grimball, 1945; Tanaka et al., 1995). But, the quantitative genetics of watermelon FSI variations have been similarly stated (Gusmini and Wehner, 2005; Gusmini and Wehner, 2007; Kumar and Wehner, 2013), e.g., the genetic locus “ClFSI-3.2” harboring the *O* gene has been significantly validated with the homologous *SUN* gene in tomato (Dou et al., 2018b). Recently, a newly identified allelic fashion revealed that the rounded watermelon shape is produced by the deletion of a 159 bp region in the CDS coding sequence of the *Cla011257* gene (Maragal et al., 2019; Legendre et al., 2020). In our study, we also identified few more genes for FL, FW, and FSI, but strong validation should be required for validation.

Fruit rind thickness (FRT) is mainly associated with resistance or susceptibility to splitting/cracking (Fan et al., 2000; Liao et al., 2019; Yang et al., 2021). Until now, molecular basis studies of watermelon fruit rind thickness have received little attention, and few genetic mapping studies have been conducted. In the recent molecular study of watermelon (Yang et al., 2021), a single major QTL of rind thickness (RTH-2.1) was mapped on Chr-02, which explained 14.74% of the phenotypic variations for the fruits with less rind thickness and signified the allelic dominance of the parental line with less rind thickness. These results were in line with the earlier published results, where genetic segregation analysis exhibited that the rind thickness is controlled by a major-effect locus positioned on Chr-09 (Fan et al., 2000). But in this study, we mapped a single major QTL (FRT-4.1) to the genetic location of Chr-04. This explained the 14.74% phenotypic variation for fruits with thinner rinds (**Figure 3**; **Table 2**).

As far as we know, our identified QTL region of FRT contradicted to the earlier reported studies (**Supplementary Table S9**), thus exhibiting the novel genetic loci controlling fruit rind hardness. Further, a frequent uniform distribution of genetic segregation was observed in fruits of F_2:3_ plant families (**Figure 6**). In the other previous study, extremely significant and positive associations were observed for individual fruit weight, cracking, and rind thickness of cherry fruit (Yamaguchi et al., 2014). Brinjal fruit rinds with high firmness showed suitable resistance to fruit cracking due to their thicker peels and affected the prolonged storage and shelf life (Liu et al., 2007). Regarding the genetically and physiologically understandings, it was reported that differential watermelon rind thickness level is similarly interconnected with reliable rind hardness, cracking resistance, and susceptibility (Li et al., 2016; Liao et al., 2019), as well as fruit weight (Yang et al., 2021). It has recently been reported that fruits with rind thickness variations bear dissimilar types of cell size and shapes, particularly due to the presence and absence of lignin accumulation in the rind cell walls (Gao et al., 2013; Yang et al., 2021). However, it was stated that class III peroxidase genes are primarily responsible for regulating the internal structure of lignin accumulation and cell wall structure (Yang et al., 2022).

Fruit flesh firmness (FFF) is a primary attribute of the premium quality and shelf life of edible fruits. A significant changes in flesh firmness involves a series of natural and complex physiological changes that trigger the metabolism of cell wall, cellulose, hemicellulose, and pectin (Brummell et al., 1999; Girard et al., 2012; Shi et al., 2013; Yoko et al., 2014; Sun et al., 2020), and mainly regulated by numerous genes and metabolic networks (Brummell et al., 1999; Brummell and Harpster, 2001; Liao et al., 2019). In contrast, fruit flesh softening (loss of firmness) is the ultimate effect of the respiratory process and ethylene bio-synthesis factor (Liao et al., 2019). Until now, few genetic mapping studies effectively mapped the major locus of regulating the flesh firmness in watermelon fruit. Juarez et al. (2013) developed novel SNP markers, constructed a genetic linkage map using the biparental F_2_ generation, and discovered a major QTL region of controlling the watermelon flesh firmness in the 9^th^ linkage group (LG). Liu et al. (2014) re-sequenced two comparative parental lines and identified the candidate region harboring the important genes regulating the edge flesh firmness on the 9^th^ LG of the watermelon linkage map, using a derived F_2_ plant population. Lu et al. (2016) performed molecular mapping and traced the major genes of edge flesh firmness on the 4^th^, 6^th^, and 8^th^ LGs of watermelon. Gao et al. (2016) used simple sequence repeat markers and a closely linked QTL marker to identify the gene controlling the firmness of watermelon flesh. Gao (2018) performed primary genetic mapping and detected a physical interval of 4.7 Mb, harboring a potential gene for controlling watermelon flesh firmness. Sun et al. (2020) used a rapid method of BSA-sequencing and preliminary mapped the two genetic regions (1.53 Mb on Chr-02 and 195 kb on Chr-08) for regulating flesh firmness. In the current study, one minor-effect QTL (FFF-11.1) was mapped on Chr-11, which explained an individual genetic effect with 9.01% PVE for less firmness of fruit flesh (**Table 2**), with uniform segregation (**Figure 6**). The physical position of this QTL was located between the 2340516~3081651 bp, exhibiting a total of 741.14 kb interval (**Figure 3**). We also noticed that our identified QTL results are inconsistent with the previously published research, as shown in **Supplementary Table S9**, respectively.

For the genetic understanding of flesh firmness regulation, it was speculated that flesh textural properties are controlled by polygonal architecture, which is the interconnected activities of proteins encoding cell-wall transformation. In watermelon, the transcription factor (MADS-box) is significantly involved in the biological processes of the transformation of plants at vegetative and reproductive growth stages “photoperiodism, pollen development, and floral organs formation, photosynthesis and nutrient metabolism, fruit development stages, maturation stages, and hormonal signal transduction pathways” (Hu et al., 2005; Liu et al., 2010; Huang et al., 2012; Li et al., 2015; Sun et al., 2020). The endogenous crude fiber and pectin content were reported as the main reasons for flesh firmness variations among the botanical groups of wild-type as well as cultivated watermelon, but the metabolic pathway differentiation of pectin and crude fiber might be a fundamental reason for divergent flesh firmness during watermelon domestication (Liu et al., 2013). It was similarly shown that the breakdown of cell walls is tightly connected with the genetic factor of fruit softening due to the ethylene-dependent accumulation of sucrose, which is regulated by the *CmMYB113* factor in melon (Gao et al., 2021). Furthermore, genes encoding glyoxysomal malate synthase, β-D-xylosidase, chloroplastic anthranilate phosphoribosyltransferase (*MELO3C011963*), and histidine kinase (*MELO3C020055*) were discovered to be involved in regulating flesh firmness in the natural population of melon (Nimmakayala et al., 2016).

Watermelon fruit is primarily consumed due to its high Brix content and health benefits (Zhang et al., 2006). The major genetic loci (QTLs) of Brix% have been identified in a few earlier studies; e.g., three minor but consistent QTLs were identified on Chr-08 that accounted for 6.87%, 5.14%, and 5.27% PVE (Sandlin et al., 2012; Liu et al., 2015), and two QTLs exhibited the main loci controlling gene expression for Brix% (Guo et al., 2006). In a recent genetic mapping study, two significant co-localized QTLs (BCC-2.1 and BCC-5.1) were identified for center flesh Brix% and three co-localized QTLs (BCE-2.1, BCE-2.2, and BCE-5.1) were identified for Brix% in the edge part of the flesh (Liang et al., 2022). The detected QTLs tightly shared the mutual genetic contributions and suggested that there might be a single locus for regulating the Brix% in the whole fruit flesh. Even though the Brix QTL (BRX-2.1) has been found on Chr-02 between 17,657,266 and 18,454,759 bp and shown to be a stable QTL with a strong genetic effect on the Brix% value (Sandlin et al., 2012; Ren et al., 2014; Ren et al., 2018). In this study, we found one major QTL (FBR-10.1), which was mapped at a 890.74 kb genetic interval on Chr-10, which explained the individual phenotypic effect with 13.98% PVE for Brix% (**Table 2**; **Figure 3**), and a transgressive segregation of FL and Brix% in fruits of F_2:3_ families (**Figure 6**) signified that elongated oblong-shaped fruits have a higher Brix% value than small rounded fruits. Overall, our detected QTLs were traced back to previously reported chromosomal regions, possibly indicating a new genomic region for sugar level regulation and biosynthesis due to the divergent genetic backgrounds of watermelon cultivars. For the genetic regulation, it was determined that *Cla000264* “*ClTST2*” is the candidate gene regulating Brix% value (Ren et al., 2018). Hence, our mapped QTL regions of watermelon Brix% are novel results (**Supplementary Table S9**), but delimitation of the mapped region is necessary for candidate gene validation.

Fruit flesh color (FFC) is a pivotal trait and most of the cultivated watermelon fruits display a sweet-tasting red flesh color that is reported to be dominant over the other flesh color categories due to epistatic effects and different gene expression profiling for different pigment synthesis (Henderson et al., 1998; Wehner, 2007; Cazzonelli and Pogson, 2010; Bang et al., 2010; Nakkanong et al., 2012; Grassi et al., 2013; Liu et al., 2015; Lv et al., 2015; Wang et al., 2019; Fang et al., 2022). The genetic locus of controlling red flesh color was mapped on Chr-02 and Chr-08 of watermelon, using a comprehensive linkage map (Hashizume et al., 2003). Then, a new major QTL region “FC-4.1” controlling the red flesh color was identified on Chr-04, with 34.68% PVE (Liu et al., 2015). This QTL was mainly associated with endogenous accumulation of lycopene content. Furthermore, an in-depth genetic mapping study validated the QTL “FC-4.1” and explained the 392,077-bp region on Chr-04, controlling the dominant red flesh color in a six-generation population of watermelon (Wang et al., 2019). Another major effect QTL “FC-10.1” region (about 519 kb) was mapped on Chr-10, which signified the regulation of differentiated pale green flesh colors associated with the major accumulation of endogenous green chlorophyll content in the flesh (Pei et al., 2021). Recently, a QTL mapping study effectively pinpointed the main genetic locus (645-kb interval) locus on Chr-04, controlling the FFC in F_2:3_ mapping population (Liang et al., 2022). In our study, we mapped just one minor QTL (FFC-5.1) at a wide chromosomal region of Chr-05 that explained the individual genetic effect with 7.42% PVE (**Table 2**; **Figure 3**), and this QTL was situated at the physical positions of 28704441~32310869 bp, exhibiting the big genetic interval (3.61 Mb). This mapped locus result was noticed as consistent with et al. (2003) but inconsistent with other published results as **Supplementary Table S9**, respectively.

The key gene for red flesh color in watermelon is *ClLCYB*, which is annotated as lycopene beta-cyclase, was finely mapped on Chr-04 using three genetic populations. In addition, two SNP non-synonymous mutations were found in the coding region of *ClLCYB* among red flesh and yellow flesh watermelon accessions (Liu et al., 2015; Wang et al., 2019). Regarding the genetic regulatory mechanism, it has been reported in many studies that the *LCYB* gene (*Cla97C04G070940*) exhibits a relative expression profiling for differentiating the pink to red flesh color gradients (Gusmini and Wehner, 2006; Perkins-Veazie et al., 2006; Bang et al., 2007; Bang et al., 2010; Kang et al., 2010; Guo et al., 2019; Wang et al., 2019). Two flesh color QTLs were identified on Chr-02 and Chr-04, and map-based cloning was performed based on the white-fleshed line and red-fleshed line. The *Cla005011* gene was considered a lycopene β-cyclase (*LCYB*) and candidate gene in the genomic region of Chr-04, and another gene was narrowed down to a region of 1,200 kb on Chr-02 (Zhang et al., 2014). Subsequently, Zhang and colleagues conducted cloning and transgenic analyses of the *LCYB* gene, and the findings revealed that the abundance of the *ClLCYB* protein rather than the *ClLCYB* transcription level was negatively correlated with lycopene accumulation (Zhang et al., 2020). *ClPHT4;2* (annotated as a function of the chromoplast-localized phosphate transporter) determines the development of flesh color through carotenoid accumulation, and it also controls the level of sweetness, which is regulated by the transcription factors *ClbZIP1* and *ClbZIP2* (Zhang et al., 2017). A single dominant gene, *Y^scr^*, was suggested to produce the scarlet red flesh color rather than the coral red flesh color (Gusmini and Wehner, 2006). Another genetic study also revealed that *Y^scr^* produces the scarlet red flesh color and was fine-mapped in a 150-Kb region on Chr-06 (Li et al., 2020). Branham et al. (2017) mapped a major QTL (FC.1) associated with β-carotene accumulation for orange flesh on Chr-01 (2.4 Mb interval). *PSY* gene expression profiling regulate the phytoene synthase which may cause the palered and orangeyellow flesh colors to occur in response to maximum endogenous carotenoids synthesis (Guo et al., 2015; Branham et al., 2017; Fang et al., 2022), and *Cla97C10G185970* was recently annotated as a plastid lipid-associated protein for regulating the differentiated pale green flesh color (Pei et al., 2021). However, in our study, we observed the contrasted minor QTL region at Chr-5, that is located at somewhat big genetic interval (**Figure 3**) that strongly needs to be delimited for identifying the candidate genes for regulating the differentiated pale green flesh color variants.

Fruit rind color (FRC) is a significant factor for evaluating the mature quality of watermelon fruit. The rinds of most commercial/edible watermelons are solid-green (G), light-green (G), or yellow (go). It has been reported that *G* > *gs* > *g* (dominant to recessive) has the allelic relationships of the traits for rind patterns (Weetman, 1937; Porter, 1937; Poole, 1944; Barham, 1956; Henderson, 1989; Henderson, 1991; Henderson, 1992; Rhodes and Zhang, 1995; Rhodes and Dane, 1999; Guner and Wehner, 2004; Kumar and Wehner, 2011). The solid-green rind was identified as completely dominant over the striped light green (*gs*) and partially dominant over the unique type of light-green, gray or yellowish-green (Johnson and Buckley, 1991; Yang et al., 2015; Kim et al., 2015). Some molecular mapping studies identified a stable major-effect genetic region located on Chr-08, controlling the watermelon fruit rind color (Park et al., 2016; Li et al., 2019). In this QTL mapping study, one major QTL (FRC-2.1) was mapped at the bottom end of Chr-02 that explained an individual genetic effect of 15.12% PVE, and the physical positions (32909754~34162281 bp) exhibited a 1.25 Mb interval (**Figure 3**). Regarding the genetic regulation, a few genetic studies revealed that watermelon fruit rind coloration is controlled by just a single gene that is located on Chr-08; however, dark-green rinds represented dominant genetic effects over light-green rinds (Park et al., 2016; Li et al., 2018). It was differentially stated that a potential gene (*ClCG08G017810* “*ClCGMenG*”) is situated on Chr-08, encoding the 2-phytyl-1,4-beta-naphthoquinone methyltransferase (Li et al., 2019). The chromosomal position of Chr-08 was traced with approximately a 262 kb of deletion in the genome assembly of watermelon (97103, v1.0) (Li et al., 2019); but, the identified gene “*ClCGMenG*” position of FFC was differentially located within 29,869,645 to 29,901,009 bp at Chr-08 of the improved genome “Charleston Gray”, and exhibited an additional 34 genes. Furthermore, a recent study mapped the FRC regulating locus was positioned from 24,184, 248 to 24,644,537 bp between adjacent SNP markers at the bottom end of Chr-08 (Liang et al., 2022). Thus, our genetic mapping results are inconsistent with the abovementioned studies (**Supplementary Table S9**), and the genetic location controlling the rind coloration needs to be narrowed down for gene annotation and functional validation.

Many watermelon cultivars possess varying degrees of fruit rind stripes (FRS) with narrow, wide, wavy and blotchy patterns (Yang et al., 2015; Maragal et al., 2022). Many QTL studies have been performed by using the developed biparental mapping populations (F_2_ and F_3_) and recombinant inbred lines. These studies identified that the differentiated watermelon rind strip pattern is generally controlled by a stable major effect genetic locus positioned on Chr-06 (Park et al., 2016; Zhang et al., 2018; Wu et al., 2019; Guo et al., 2019; Yue et al., 2021; Wang et al., 2022; Liang et al., 2022) and Chr-09 (Maragal et al., 2022), except the minor variations in genetic positions found in this study. Our genetic mapping study exhibited the contrasted QTL results as compared to the earlier reported QTLs. Herein, one minor QTL (FRS-4.1) was mapped at the start end of Chr-04 that explained an individual genetic effect of 9.75% PVE. The physical positions (2031314~2731617 bp) exhibited a 700.30 kb interval (**Figure 3**) that depicted a total of 16 putative genes.

For the genetic regulation, it was first reported that the single gene locus “*S*” mainly controls the watermelon FRS (Weetman, 1937). However, FRS regulation also depends upon the divergences in genetic background of watermelon cultivars. Some alleles at the *g* locus positioned on Chr-08 control the wide-stripe (*g^W^*), medium-stripe (*g^M^*), narrow-stripe (*g^N^*), and solid-light green or light-green (*g*), with a dominance order of *G* > *g^W^* > *g^M^* > *g^N^* > *g* (Kim et al., 2015; Lou and Wehner, 2016; Zhang et al., 2018). In a recent study, linkage mapping was done by using the two differentially derived mapping populations and novel stable QTLs/genes regions were spotted on Chr-09. The comparative genomic analysis revealed two candidate genes “*Cla97C09G175170* and *Cla97C09G175150*” regulating the blotchy stripes and wavy type rind stripe pattern, and sequence analysis of the *Cla97C09G175170* gene exhibited the 3 bp deletion on the 11^th^ exon associated with strip color (Maragal et al., 2022). To the best of our knowledge, the genetic locus (FRS-4.1) for wavy type rind stipes trait has been identified for the first time in our study (**Supplementary Table S9**), but more molecular research is needed to further clarify the results variations and identify gene homologs contributing to differentiating the rind stripe of watermelon.

### Seed QTLs

Seed is an integral part of the plant life cycle and a significant determinant of growth and development. Seed genetics have been extensively studied in major food crops that are consumed directly or indirectly as grains or seeds, such as rice, wheat, and soybean, but few genetic studies are available for vegetable crops (Amanullah et al., 2021). In watermelon, a wide range of seed size variation is present among the various cultivars (Guo et al., 2020). The inheritance of seed size/shape was first reported, and small-to-large sized seeds were found to be dominant over medium-sized seeds (Poole et al., 1941). Later, the quantitative nature of seed size/shape variation was further observed in a few studies.

Until now, a total of 61 QTLs (twenty seed length QTLs, nineteen seed width QTLs, 3 seed thickness QTLs, and nineteen QTLs of seed weight) have been identified with major variations in PVE% across the different chromosomal positions of the whole genome chromosome of watermelon “Chr-01, Chr-03, Chr-06, Chr-07, Chr-10, LG-04, and LG-09” using numerous mapping populations derived from crossing of wild type and cultivar parent lines (Prothro et al., 2012a; Prothro et al., 2012b; Sandlin et al., 2012; Ren et al., 2012; Meru and McGregor, 2013; Liu et al., 2014; Kim et al., 2015; Zhou et al., 2016; Li et al., 2018; Meru et al., 2018; Luan et al., 2019; Guo et al., 2020; Li et al., 2021; Osae et al., 2021; Liang et al., 2022). In this study, three major QTLs of SWt (SWt-4.1, SWt-5.1, and SWt-10.1), two co-localized QTLs of STh and SL (STh-7.1 & SL-7.1), two major QTLs of SW (SW-4.1 and SW-10.1), one major QTL (SSI-6.1), and one major QTL (SCT-1.1) were detected on six differential chromosomes (Chr-01, Chr-04, Chr-05, Chr-06, Chr-07, and Chr-10) (**Table 2**; **Figure 3**). The QTL results of Chr-04, Chr-06, Chr-7, and Chr11 are in line with the earlier published QTL results of the above mentioned studies; but, we observed that the mapped QTL of seed weight (SWt-5.1) is a novel QTL because it wasn’t reported earlier (**Supplementary Table S9**). Further, our identified co-QTLs of seed width, seed length, and seed thickness traits on Chr-07 and Chr-10 also signified the stable and major genetic effects. These QTLs were also consistent and supported the contribution of uniform distribution of segregation also displayed the mutual quantitative inheritance of comparative watermelon parent lines within seeds of F_2:3_ families (**Figure 7**).

Regarding the understanding of seed genetic mechanisms, it has been stated that the large-to-medium length of seed is controlled by two major genes (*l* and *s*), and small-sized seeds seem dominant over medium-sized seeds. Later, two genes (*Ti* for tiny seed and *ts* for tomato seed) were also reported for small-sized seeds (Poole et al., 1941; Zhang et al., 1994; Tanaka et al., 1995; Zhang, 1996). Later, Zhang and Zhang (2011) reported that a recessive gene pair significantly determines the size of seed, but additional modifiers are also involved in the seed size regulations. Watermelon seed size was considered as a quality trait and supposed to be controlled by a single dominant gene (Kim et al., 2015). Fine genetic mapping revealed the major-effect locus of controlling the seed size/shape “qSS-6.1”, harboring a total of three candidate genes (*Cla009291*, *Cla009301*, and *Cla009310*) (Li et al., 2018). The *Cla009291* gene was exhibited as encoding the MDR protein “mdtK” and was differentially expressed in the seed development stages of large and small seeded lines. *The Cla009310* gene exhibited a major SNP in the 1^st^ exon region and was presumed to be a candidate gene that encodes an unknown protein for regulating the seed size/shape between comparative watermelon lines. Further, *Cla009301* was found as the homolog of SRS3 (SMALL AND ROUND SEED) for a BY-kinesin-like protein, known as the seed size regulator in rice (Kanako et al., 2010). Further, the relative expression profiling of the *Cla007520* gene was noticed to be higher at the early stage of seed formation, but the *Cla007520* gene seemed to belong to the CPP protein family and its related amino acid components were similar to those of the *AtTSO1*, responsible for promoting cell proliferation initiation during ovule development (Luan et al., 2019). However, seed size and shape, as well as their associated traits, are elastic in nature and change in different environmental locations (Fisher et al., 2017). Thus, our reported seed-related QTL segments might harbor some potential genes that would exhibit significant expression profiling but need to be narrowed down, respectively. Furthermore, the identified genes of all traits were categorized into the molecular function, biological process, cellular components, and GO terms with significant enrichment (**Figure 9**; **Supplementary Tables S7**,**S8**). There were a few important GO enrichment terms and KEGG pathways which signified the polygenic phenomenon for regulatory mechanisms of ovary, fruit, and seed phenotypes. In addition, some explicit genes were also indicating the same regulatory pathways for different traits, and those genes were presented in tightly co-localized QTL regions with high LOD scores and major PVE%. A comprehensive gene descriptions have been reported in our current study but there is still no direct evidence about stable QTLs/genes controlling these traits of watermelon due to extreme divergences in genetic backgrounds.

In summary, we assumed that our identified genes might harbor expression profiling for the genetic regulation of watermelon phenotypes, but gene functional validation is necessary based on fine genetic mapping of the mapped QTL region. Further, our constructed linkage map and mapped QTL regions will provide an important genetic basis for comparative genetic mapping and marker-assisted selection (MAS) in watermelon.

## Data availability statement

The datasets presented in this study can be found in online repositories. The names of the repository/repositories and accession number(s) can be found below: https://www.ncbi.nlm.nih.gov/, PRJNA878948.

## Author contributions

SA designed and completed the molecular genetics and breeding experiments, did the formal analysis, drafted the manuscript, and reviewed & edited the manuscript. SL helped in performing the formal analysis. BO, TY participated in the field sampling. FA assisted the theoretical guidance. XW provided the seeds of the watermelon parent lines. MG assisted in the practical guidance. HL, PG and FL supervised the research project, reviewed & edited the manuscript. All authors contributed to the article and approved the submitted version.
