# Supplementary material for: Primary mapping of quantitative trait loci regulating multivariate horticultural phenotypes of watermelon (Citrullus lanatus L.)
Source: Front Plant Sci. 2023 Jan 12;13:1034952. doi: 10.3389/fpls.2022.1034952 (PMC9877429; doi:10.3389/fpls.2022.1034952)
Supplement: Supplementary file 1 [file DataSheet_1.zip › Supplementary Material/Supplementary Figure 1-2.docx]

**Supplementary Figures**


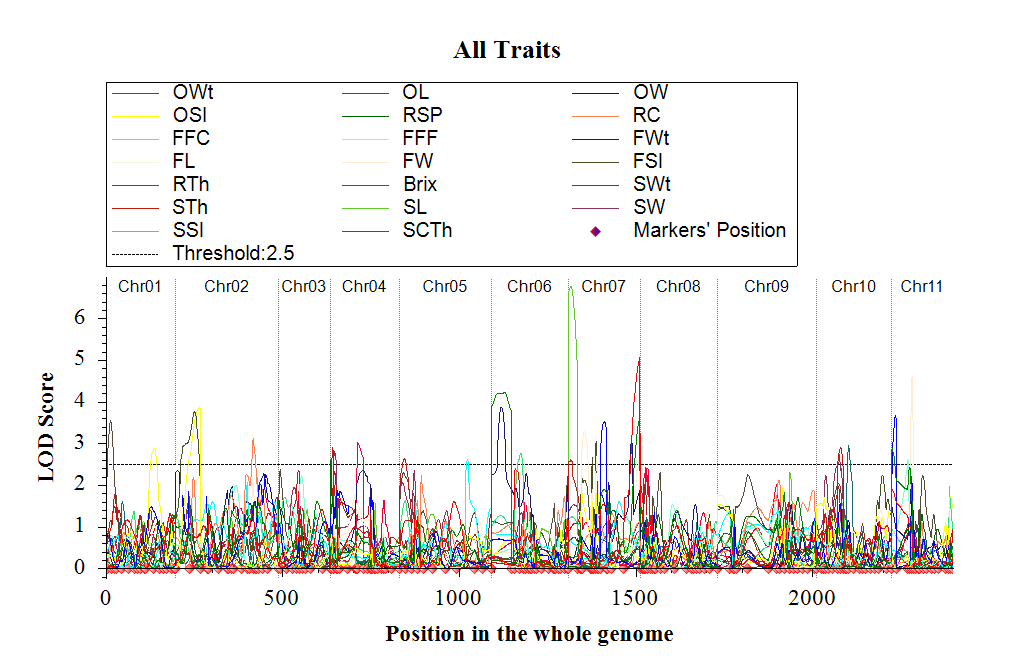


**Supplementary Figure S1 |** The LOD score distribution plot for QTL mapping of all trait.


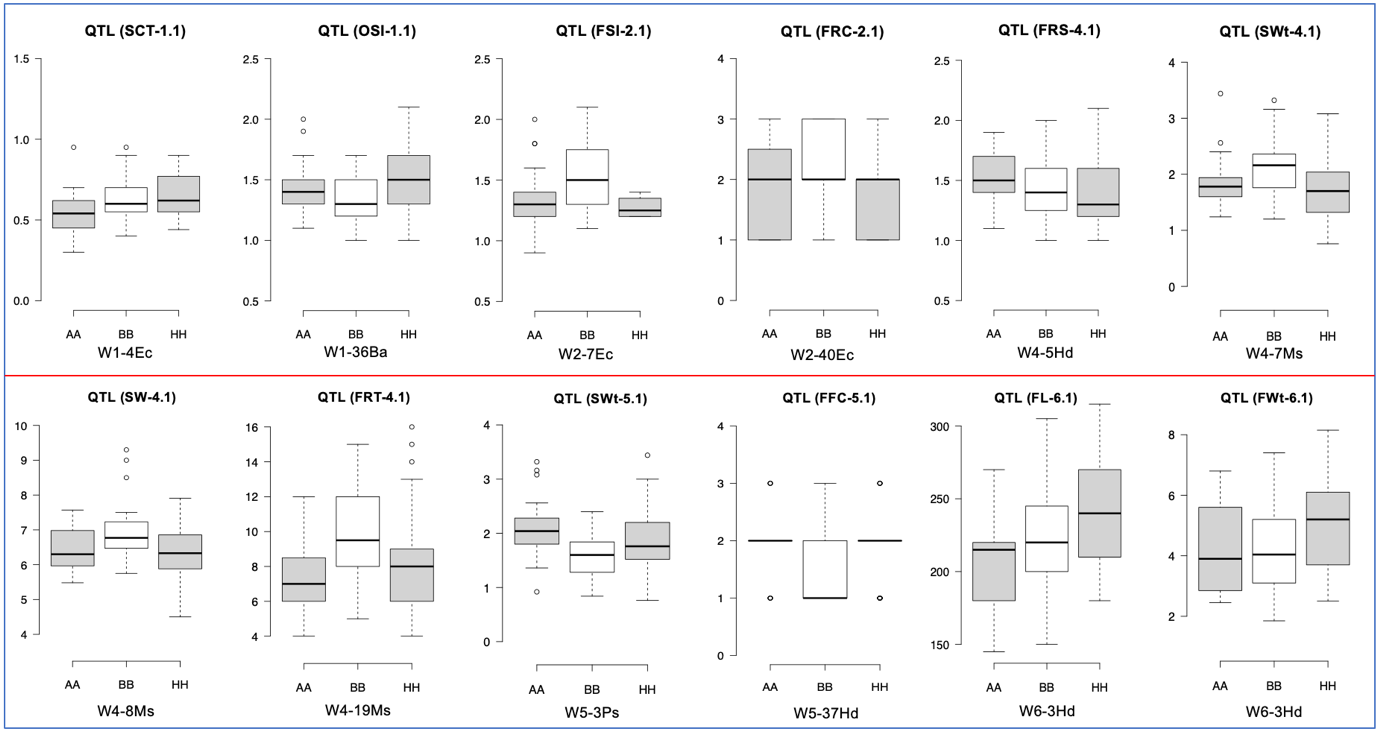


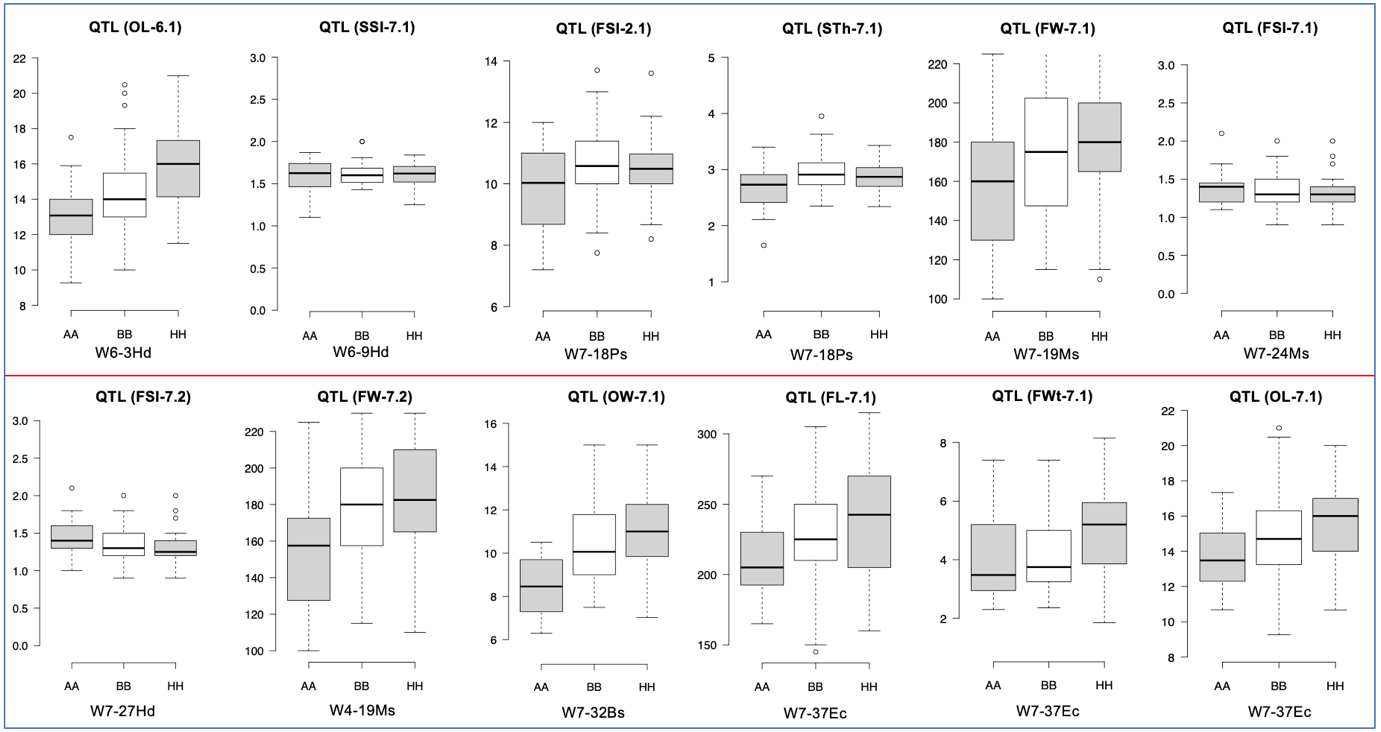


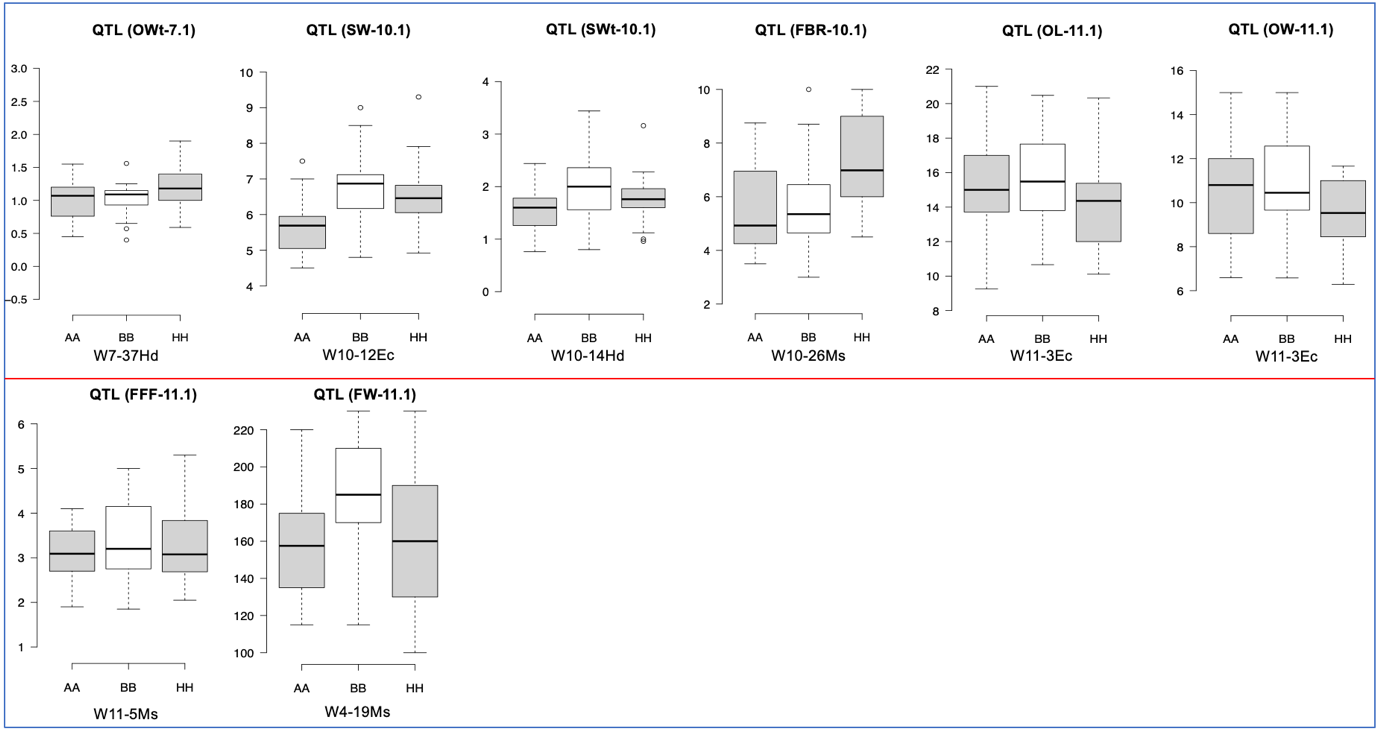


**Supplementary Figure S2 |** Box plots of SNP allelic effects underlying identified QTLs of ovary, fruit, and seed traits of watermelon.
